# Chaperone assisted recombinant expression of a mycobacterial aminoacylase in *Vibrio natriegens* and *Escherichia coli* capable of N-lauroyl-L-amino acid synthesis

**DOI:** 10.1186/s12934-023-02079-1

**Published:** 2023-04-21

**Authors:** Gerrit Haeger, Jessika Wirges, Nicole Tanzmann, Sven Oyen, Tristan Jolmes, Karl-Erich Jaeger, Ulrich Schörken, Johannes Bongaerts, Petra Siegert

**Affiliations:** 1 Institute of Nano- and Biotechnologies, Aachen University of Applied Sciences, 52428 Jülich, Germany; 2 TH Köln - Campus Leverkusen, 51379 Leverkusen, Germany; 3 Institute of Molecular Enzyme Technology, Heinrich Heine University Düsseldorf, 52425 Jülich, Germany; 4 Institute of Bio- and Geosciences IBG-1: Biotechnology, Forschungszentrum Jülich GmbH, Jülich, 52425 Germany

**Keywords:** Aminoacylase, *Vibrio natriegens*, Chaperone co-expression, Inclusion bodies, Acyl-amino acids

## Abstract

**Background:**

Aminoacylases are highly promising enzymes for the green synthesis of acyl-amino acids, potentially replacing the environmentally harmful Schotten-Baumann reaction. Long-chain acyl-amino acids can serve as strong surfactants and emulsifiers, with application in cosmetic industries. Heterologous expression of these enzymes, however, is often hampered, limiting their use in industrial processes.

**Results:**

We identified a novel mycobacterial aminoacylase gene from *Mycolicibacterium smegmatis* MKD 8, cloned and expressed it in *Escherichia coli* and *Vibrio natriegens* using the T7 overexpression system. The recombinant enzyme was prone to aggregate as inclusion bodies, and while *V. natriegens* Vmax™ could produce soluble aminoacylase upon induction with isopropyl β-d-1-thiogalactopyranoside (IPTG), *E. coli* BL21 (DE3) needed autoinduction with lactose to produce soluble recombinant protein. We successfully conducted a chaperone co-expression study in both organisms to further enhance aminoacylase production and found that overexpression of chaperones GroEL/S enhanced aminoacylase activity in the cell-free extract 1.8-fold in *V. natriegens* and *E. coli*. Eventually, *E. coli* ArcticExpress™ (DE3), which co-expresses cold-adapted chaperonins Cpn60/10 from *Oleispira antarctica*, cultivated at 12 °C, rendered the most suitable expression system for this aminoacylase and exhibited twice the aminoacylase activity in the cell-free extract compared to *E. coli* BL21 (DE3) with GroEL/S co-expression at 20 °C. The purified aminoacylase was characterized based on hydrolytic activities, being most stable and active at pH 7.0, with a maximum activity at 70 °C, and stability at 40 °C and pH 7.0 for 5 days. The aminoacylase strongly prefers short-chain acyl-amino acids with smaller, hydrophobic amino acid residues. Several long-chain amino acids were fairly accepted in hydrolysis as well, especially N-lauroyl-L-methionine. To initially evaluate the relevance of this aminoacylase for the synthesis of N-acyl-amino acids, we demonstrated that lauroyl-methionine can be synthesized from lauric acid and methionine in an aqueous system.

**Conclusion:**

Our results suggest that the recombinant enzyme is well suited for synthesis reactions and will thus be further investigated.

**Supplementary Information:**

The online version contains supplementary material available at 10.1186/s12934-023-02079-1.

## Background

L-Aminoacylases catalyze the hydrolysis of N-acyl-L-amino acids. The reverse reaction, namely the synthesis of acyl-amino acids from amino acids and fatty acids, is also possible. Conventionally, these molecules are produced by chemical synthesis using the Schotten-Baumann reaction, which needs to employ fatty acyl chlorides for acylation. Besides the chlorination with toxic chemicals like thionyl chloride or phosgene, sodium chloride is stoichiometrically produced as waste. Therefore, aminoacylases are highly interesting enzymes for an alternative biocatalytic, green synthesis. Among aminoacylases (EC 3.5.1.14) mostly mammalian enzymes have been studied. The porcine enzyme pAcy1 was extensively characterized regarding mechanism and mode of action [1, 2], recombinant expression [3] and synthesis of acyl-amino acids [4–6]. The industrial application is hampered by substrate specificity, difficult expression and insufficient stability. The fungal aminoacylases, sourced from *Aspergillus melleus* or *A. oryzae*, in contrary, have a long-established biocatalytic application. However, they are primarily used for the production of enantiomerically pure L-amino acids from a racemic mixture of acetylated amino acids, rather than for the synthesis of acyl-amino acids. Aminoacylases for the synthesis of acyl-amino acids should accept long acyl chains as substrates, so that amino acid-based surfactants or emulsifiers can be produced. Furthermore, the biocatalysts should be easily produced and purified and should be sufficiently stable.

Bacterial aminoacylases show more favorable characteristics for the synthesis of acyl-amino acids and have been found in various bacterial genera, among them *Bacillus* [7, 8], *Burkholderia* [9], *Corynebacterium* [9], *Pseudomonas* [10], or *Streptomyces* [11–14]. Aminoacylases from *Streptomyces* species seem promising candidates, as multiple strains have been identified that produce enzymes with interesting properties. An alpha-aminoacylase (AA) and an epsilon-lysine-acylase (ELA) have been described in *S. mobaraensis* (SmAA [12], SmELA [11, 15]) and *S. ambofaciens* (SamAA, SamELA [14, 16]). Especially SamAA from *S. ambofaciens* showed good catalytic potential and a broad substrate specificity [14, 17]. Besides these bacterial sources, aminoacylases have also been described from *Mycobacterium smegmatis* [18, 19] in 1964 and 1972, respectively. Protein sequences for these enzymes were not specified, and no further data regarding mycobacterial aminoacylases have been published yet. The gram-positive bacteria *Streptomyces* and *Mycobacterium* are distantly related, as they belong to the class of Actinomycetia, characterized by a high G + C content of their genomes. At first glance, members of those two genera are clearly distinct, but similarities can be found on a cellular and a molecular level [20]. A unique characteristic of *Mycobacteria* is the occurrence of extremely long-chain mycolic acids that form a waxy layer in the bacterial cell wall suggesting that these organisms may possess aminoacylases which accept long-chain acyl-amino acids.

For characterization and evaluation of the potential in synthesis of acyl-amino acids, it is advantageous to heterologously overexpress the relevant aminoacylase. This way, enough protein of interest can be obtained. By fusing the aminoacylase gene to an affinity tag, purification is straightforward. In most cases, *E. coli* is used as an expression host strain [21], however, problems may occur including inclusion body (IB) formation. This is particularly relevant if the T7 expression system is used [22]. To prevent this, the *E. coli* Tuner™ strain (Novagen), a *lacY* deletion mutant that allows to adjust the cellular IPTG concentration, has been constructed [23]. Another option is the usage of autoinduction media that contain glucose and lactose in which bacteria first grow to high densities before expression is induced [24].

Another approach to increase the solubility of aggregation-prone recombinant proteins is the co-expression of molecular chaperones, which can help to fold otherwise misfolded proteins [25]. Most commonly, combinations of so-called heat-shock proteins like GroEL/GroES and DnaK/DnaJ/GrpE, or foldases like Trigger Factor (Tf) can be co-expressed. Since these chaperones function differently, positive influence on recombinant proteins must be tested individually or from combinations thereof. Trigger Factor is an ATP-independent chaperone with additional peptidylprolyl isomerase activity that binds to nascent polypeptide chains at ribosomes and is thus the first chaperone to interact with newly synthesized proteins. It mediates first folding steps, can prevent premature folding and restricts access of downstream chaperones or can prevent premature degradation [26]. The chaperones DnaK, DnaJ and GrpE function in concert and should thus be co-expressed, as overexpression of DnaK or DnaJ alone can impair cell viability. The ATP-dependent DnaK is responsible for main folding activity and can work both co- and post-translationally, and DnaJ and GrpE modulate ATP hydrolysis and conformational changes [25]. The chaperonine GroEL/GroES is a multimeric protein that forms a barrel-like structure composed of two heptameric rings of GroEL which can be closed by heptameric rings of GroES on each side, forming a lid. Upon entering of the misfolded protein into the cavity formed by the GroEL ring, the lid formed by GroES closes and the protein substrate gets folded by conformational change directed by hydrolysis of ATP [27]. As an alternative expression host, we have investigated *Vibrio natriegens*, a marine bacterium which has recently gathered attention for its use in biotechnology due to its extraordinary high growth rates. Its close genetic proximity to *E. coli* renders many molecular biology tools applicable also for *V. natriegens*, as shown for the plasmid pET-based expression system, making this strain an interesting alternative to *E. coli* for heterologous protein expression [28, 29].

Here we present the characterization of a novel mycobacterial aminoacylase and show its general applicability in the synthesis of acyl-amino acids. The recombinant expression by classical IPTG-induction in *E. coli* BL21 (DE3) led to inclusion body formation without obtaining soluble protein. We improved the expression by changing the mode of induction, co-expression of molecular chaperones, and adjusting expression temperatures. Furthermore, expression in *V. natriegens* with chaperone co-expression was established.

## Results and discussion

### Cloning and sequence analysis of the ***msAA*** gene from ***Mycolicibacterium smegmatis*** MKD 8

The motivation of this study was to identify a novel aminoacylase gene and facilitate its heterologous expression. The aminoacylase should have the ability to synthesize acyl-amino acids with long acyl chains and was searched in the sequence scope of *Mycobacteria*. The aminoacylase MsAA was identified by NCBI BLASTp homology search. The streptomycetal protein sequences for SmAA (Accession No. BAI44523.1) and SamAA (Accession No. AKZ54783.1) were used as baits in a BLASTp search for homologous sequences in *Mycolicibacterium smegmatis.* The search revealed a protein sequence which shows 58% similarity to both SmAA and SamAA and was designated MsAA. The encoding nucleotide sequence was deduced. The obtained DNA sequence was optimized regarding the codon usage of *E. coli* and the synthetic DNA strand was ordered. By Golden Gate cloning, the gene was cloned into pET28a expression vectors with either N- or C-terminal Strep-tag or without an affinity tag. The calculated molecular weight of MsAA is 48.5 kDa and 49.9 kDa with the affinity tag.

### Protein sequence analysis and structure prediction

**Fig. 1:**
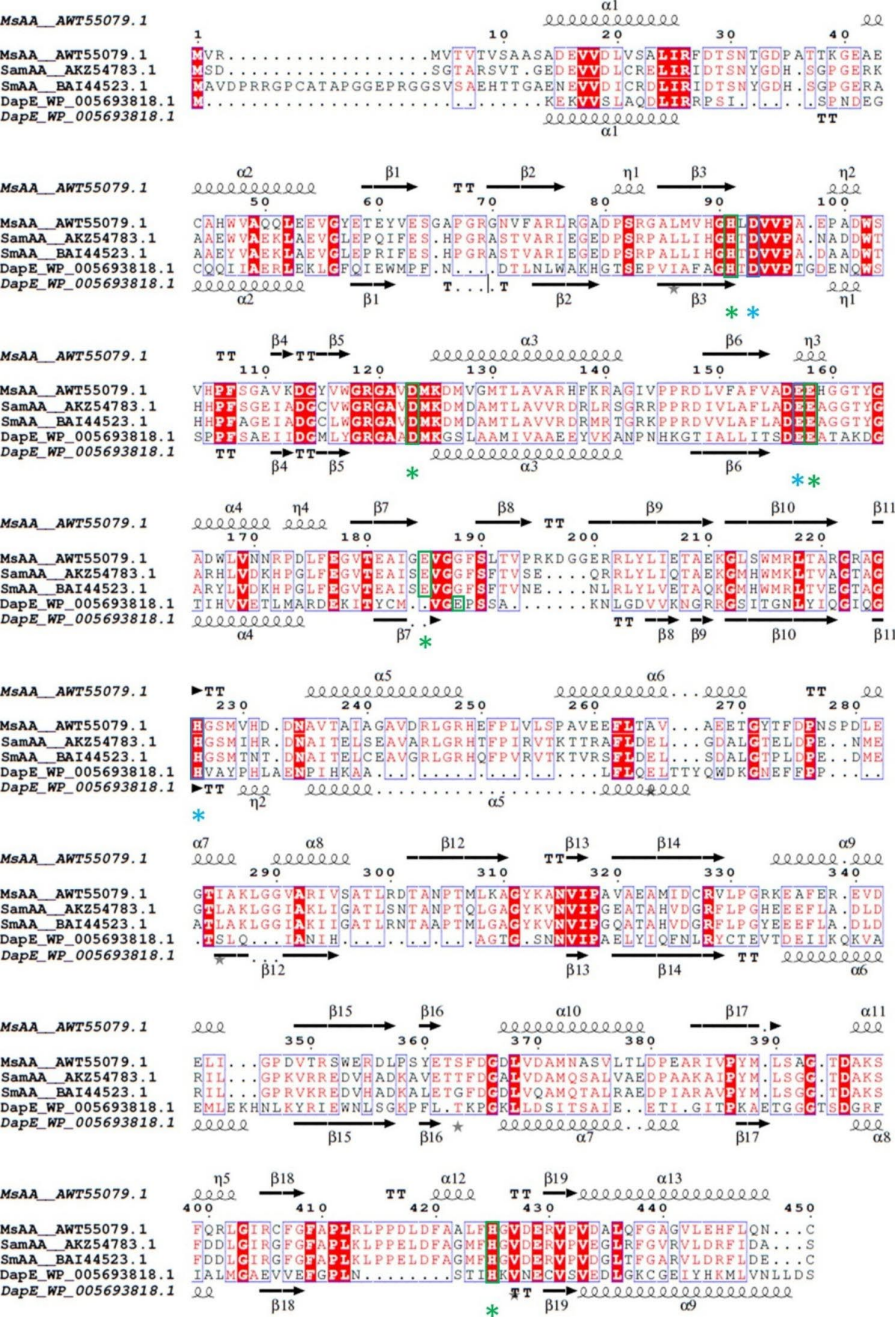
Multiple sequence alignment of MsAA and homologous proteins MsAA from *M. smegmatis* (AWT55079.1), the aminoacylases SamAA from *S. ambofaciens* (AKZ54783.1), SmAA from *S. mobaraensis* (BAI44523.1), and DapE from *Haemophilus influenzae* (WP_005693818.1) are shown. The alignment generated using T-Coffee and displayed with ESPript 3.0. The conserved metal-binding residues (H91, D123, E158, E185, H425) and catalytic residues (D93, E157, H226) are highlighted by green and blue boxes with asterisks. The annotation of the secondary structural elements shown above is based on the ColabFold-generated structure of MsAA; the secondary structural elements shown below belong to DapE (imported from PDB-entry 5vo3). The secondary structure is displayed with arrows for β-strands, squiggles for α-helices or 3_10_(η)-helices and the letters TT for turns

**Fig. 2:**
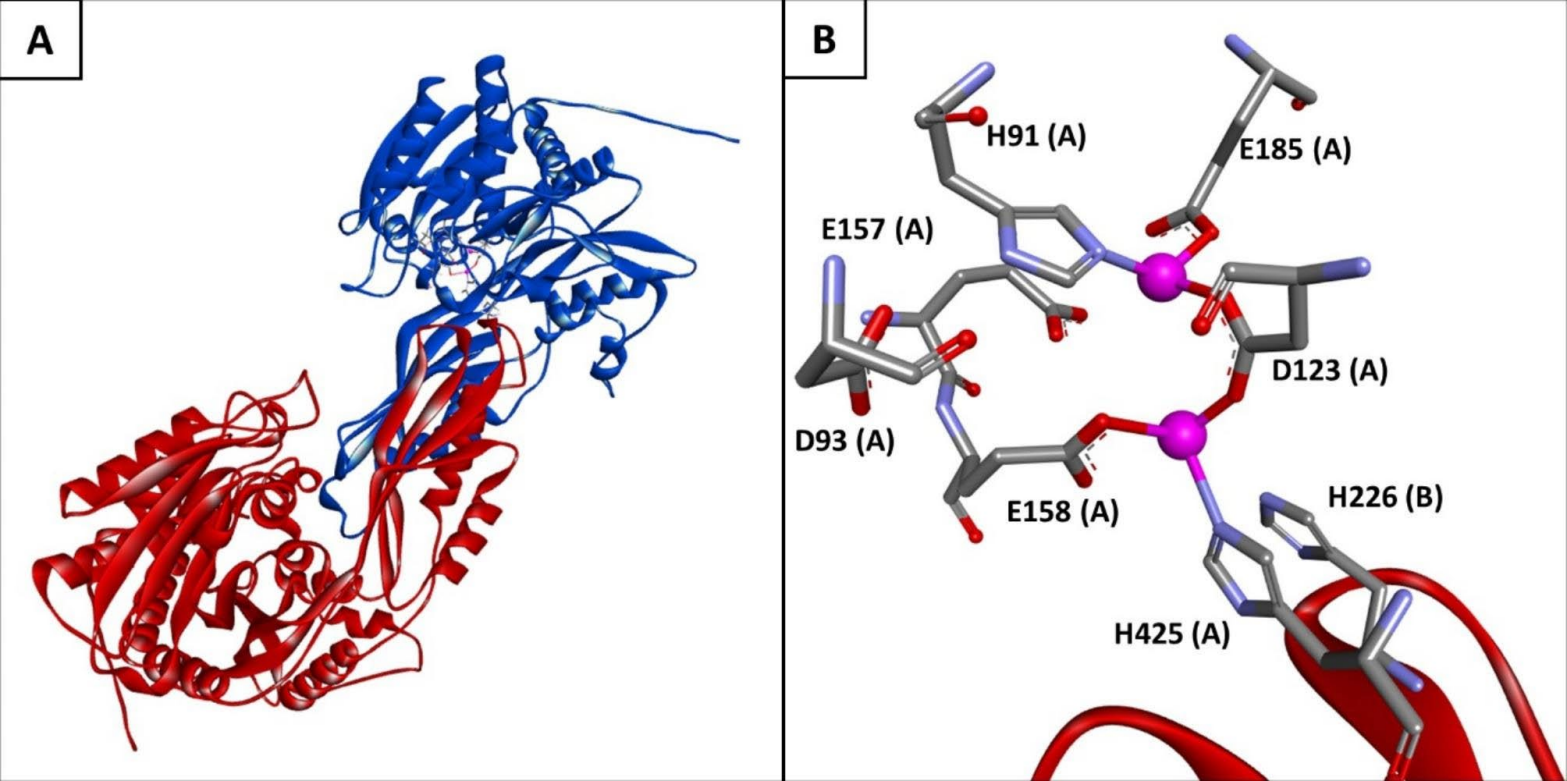
Predicted protein structure of MsAA using ColabFold2. **(A)** 3D structure with monomers shown in red and blue. **(B)** Active site of MsAA with two bound zinc ions (magenta balls) and metal-binding and catalytic residues from both dimers **(A and B)** are shown

Analysis of the protein sequence comprising 450 amino acids revealed conserved catalytic (D93, E157, H226) and metal-binding (H91, D123, E158, E185, H425) residues as well as signature motifs for the M20 peptidase family [12, 30, 31]. Members of the M20 peptidase family also include non-peptidase homologues and bind two cocatalytic zinc ions, usually bound by histidine, glutamic acid or aspartic acid [32]. The three-dimensional protein structure of dimeric MsAA was predicted with the ColabFold algorithm. In general, the generated structure showed very good per-residue confidence metric with a pLDDT of 95.2 (local accuracy) and a pTM score of 0.92 (global accuracy). The multiple sequence alignment of MsAA, SamAA, SmAA, and DapE is shown in Fig. 1, along with the secondary structural elements taken from the predicted structure of MsAA and a crystal structure from DapE. We used the MIB metal ion-binding site prediction and docking server to predict zinc binding sites based on the AlphaFold-generated structure. Two zinc-binding sites were predicted in which the zinc atoms are bound by residues which are conserved in the M20 peptidase family. The distance of metal-binding atoms and zinc ions was approximately 2 Å. The predicted structure of MsAA with its bound zinc ions is shown in Fig. 2. Structurally, members of the M20 family can either be monomeric or dimeric proteins. One domain of the structure is referred to as the catalytic domain, as it contains catalytic and metal-binding residues, while the other domain is called lid domain for monomeric or dimerization domain for dimeric enzymes. The two domains form an internal cavity harboring the active site [32]. In the case of the homologous dimeric DapE, a succinyl-diaminopimelate desuccinylase, a histidine (H194) from the dimerization domain enters the active site of the opposing monomer [31]. This histidine is also conserved in MsAA as H226 (Fig. 1). From the predicted dimeric protein structure of MsAA, the characteristic dimerization domain and catalytic domain can be distinguished (Fig. 2(A)). The active site is located in the cavity formed between the two domains. The H226 residue from one dimer reaches into the active site of the opposing dimer (Fig. 2(B)).

### Comparative expression of MsAA in ***E. coli*** and ***V. natriegens*** with co-expression of molecular chaperones and affinity purification

Expression of aminoacylase MsAA in *E. coli* BL21 (DE3) with IPTG resulted in the formation of inclusion bodies. The issue could be solved by growing the cells in autoinduction medium. This way, a fraction of soluble MsAA was obtained; however, a significant amount of insoluble enzyme still prevailed. Hence, we systematically optimized the expression conditions in *E. coli* and *V. natriegens* by varying induction mechanism, cultivation temperature and duration as well as by co-expression of molecular chaperones. To our best knowledge, this is the first study on chaperone co-expression in *V. natriegens*.

Since *V. natriegens* does not carry the genes for lactose import and catabolism, autoinduction with lactose was not possible; instead, expression was induced with IPTG. To better compare the expression potential of *E. coli* and *V. natriegens*, the *lacY*-deletion mutant *E. coli* Tuner (DE3), was investigated as well.

### Expression in Vibrio natriegens Vmax™

**Fig. 3:**
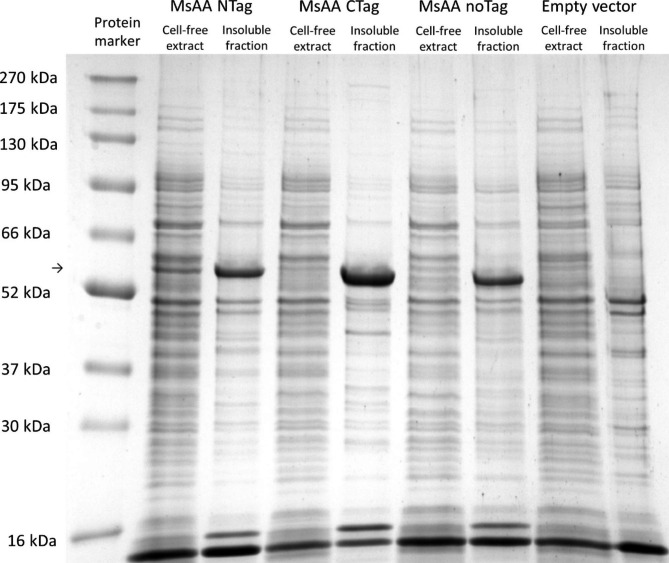
SDS-PAGE analysis of cell extracts obtained from *V. natriegens* Vmax™ after expression of MsAA variants Cells were grown in BHIv2 medium for 5 h at 30 °C. The arrow indicates the position of MsAA variants Lane 1: protein marker (BlueEasy Prestained Protein Marker, Nippon Genetics); lane 2: cell-free extract with MsAA NTag; lane 3: insoluble fraction with MsAA NTag; lane 4: cell-free extract with MsAA CTag; lane 5: insoluble fraction with MsAA CTag; lane 6: cell-free extract with MsAA noTag; lane 7: insoluble fraction with MsAA noTag; lane 8: cell-free extract of empty vector control; lane 9: insoluble fraction of empty vector control

As an alternative system for T7-based, IPTG induced expression, *V. natriegens* Vmax™ was tested for expression of MsAA. In a first step, MsAA was expressed with all Strep-tag variants, namely N-or C-terminally or without affinity tag and at 37 °C, 30 °C and 20 °C to determine the optimal expression temperature. As the N-terminally tagged MsAA was best produced in soluble form and yielded less inclusion bodies, MsAA NTag was further used in this study. Regarding temperature, expression 30 °C (Fig. 3) was found to be most suitable, as IB formation was reduced compared to 37 °C (Additional file 1: Fig. S1). At 20 °C, growth and total protein production was hampered (Additional file 1: Fig. S2). Strikingly, in contrast to *E. coli* BL21 (DE3), *V. natriegens* Vmax could form soluble and active MsAA upon induction with 1 mM IPTG. Therefore, we conducted the chaperone co-expression experiments at 30 °C cultivation temperature for 4 h.

**Fig. 4:**
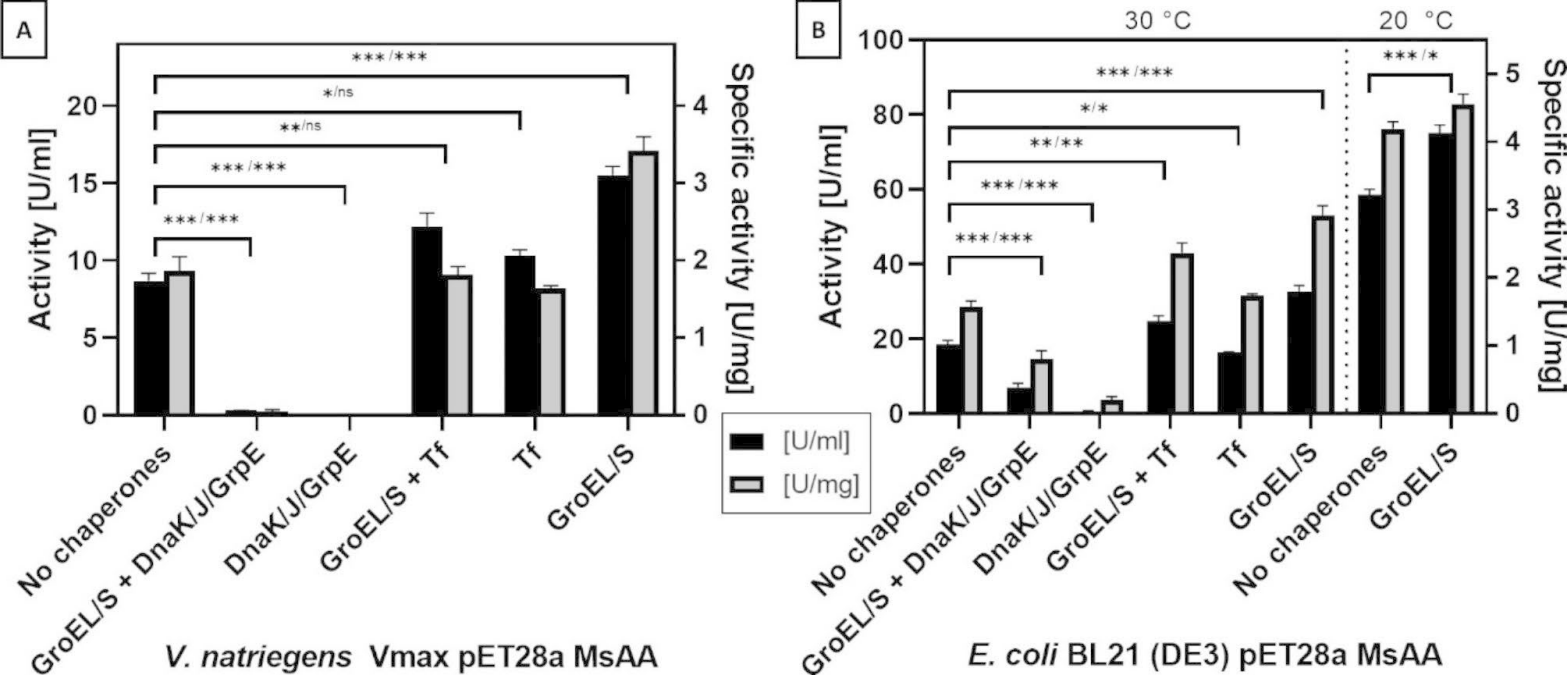
Effect of chaperone co-expression on MsAA NTag overexpression on aminoacylase activity Aminoacylase activity and specific activity were measured from the cell-free extract from *V. natriegens* Vmax™ and *E. coli* BL21 (DE3). Activity was measured in triplicates and expression without and with chaperones were repeated as three biological replicates. Statistical significance for the differences in activity and specific activity calculated by unpaired students t-test was indicated as followed: not significant (ns) for p > 0.05, * for p ≤ 0.05, ** for ≤ 0.01, and *** for p ≤ 0.001

We transformed *V. natriegens* Vmax with the chaperone plasmid set from Takara Bio containing pGKJE8 (GroEL/S, DnaK/J/GrpE), pKJE7 (DnaK/J/GrpE), pGTf2 (GroEL/S, Tf), pTf16 (Tf) or pGro7 (GroEL/S) and pET28a MsAA NTag to generate chaperone overexpression strains. Without chaperones, activity and specific activity in the cell-free extract was 8.7 (± 0.5) U/ml and 1.9 (± 0.2) U/mg, respectively. The co-expression with pGro7 lead to an almost 1.8-fold increase of activity to 15.5 (± 0.6) U/ml. Specific activity also increased 1.8-fold to 3.4 (± 0.2) U/mg. The co-expression from pGTf2 and pTf16 also raised activity, to 12.2 (± 0.9) U/ml and 10.3 (± 0.4) U/ml, respectively, while having a non-significant effect on specific activity. In contrast, pGKJE8 and pKJE7 had detrimental effects on soluble protein expression, abolishing aminoacylase activity in the cell-free extract (Fig. 4(A); gels from SDS-PAGE in Additional file 1: Fig. S3 and S4). Cultivation for further 20 h led to reduction of activity (1.6 U/ml for Vmax and 7.6 U/ml for Vmax pGro7; data not shown). We purified recombinant MsAA NTag from *V. natriegens* Vmax and *V. natriegens* Vmax pGro7 and obtained specific activities of 122.1 (± 0.5) U/mg and 129.2 (± 3.4) U/mg (results of SDS-PAGE analysis in Additional file 1: Fig. S5 and S6).

### Expression in ***E. coli*** BL21 (DE3)

With *E. coli* BL21 (DE3), *E. coli* BL21 (DE3) pGro7 and *E. coli* Tuner (*lacZY*^–^), no aminoacylase activity could be detected in the cell-free extracts upon IPTG induction, and MsAA was found only in the insoluble fractions (Additional file 1: Fig. S7 and S8). Hence, autoinduction with 0.2% lactose additionally to glucose in TB-AIM medium was performed. Growth of *E. coli* was much slower compared to *V. natriegens* Vmax, so the cells were harvested after 24 h cultivation at 30 °C. *E. coli* BL21 (DE3) was transformed with the five chaperone plasmids and all strains were used for the expression study.

The co-expression of chaperones in *E. coli* revealed results comparable to *V. natriegens* Vmax™. The co-expression of GroEL/S from pGro7 had the greatest influence on aminoacylase activity, raising activity and specific activity 1.8-fold from 18.5 (± 1.0) U/ml to 32.7 (± 1.6) U/ml and from 1.6 (± 0.1) U/mg to 2.9 (± 0.1) U/mg, respectively. Furthermore, expression of chaperones GroEL/GroES and Tf from plasmid pGTf2 was beneficial with respect to formation of soluble MsAA. Again, the co-expression of chaperones from plasmids pGKJE8 and pKJE7 resulted in lowered enzymatic activity (Fig. 4(B); SDS-PAGEs in Additional file 1: Fig. S9 and S10).

Additionally, we compared the expression with and without pGro7 at 20 °C. We found enhanced aminoacylase activity compared to expression at 30 °C in both strains with 58.6 (± 1.4) U/ml in *E. coli* BL21 (DE3) and 75.0 (± 2.2) U/ml in *E. coli* BL21 (DE3) pGro7. Hence, activity could be more than doubled by lowering the expression temperature. The influence of the chaperone co-expression on activity was less pronounced by lowering the expression temperature and led to approximately 1.3-fold enhancement. Protein formation is slower at 20 °C giving proteins more time to fold correctly; the refolding activity of GroEL/S may thus not be required. On the other hand, the chaperonine GroEL/S has its temperature optimum at 30 °C and might thus not work as efficiently at 20 °C [33]. Nonetheless, for *E. coli* BL21 (DE3), the optimized expression conditions for MsAA include co-expression of GroEL/S from pGro7 at 20 °C cultivation temperature, induction of chaperone expression with 0.5 mg/ml arabinose at the start of the cultivation in TB-AIM growth medium, autoinduction of the pET-system with 0.2% lctose and harvesting after 24 h expression. We purified recombinant MsAA from *E. coli* BL21 (DE3) and *E. coli* BL21 (DE3) pGro7 and found specific activities of 129.4 (± 0.9) U/mg and 131.0 (± 6.0) U/mg (Additional file 1: Fig. S11 and S12).

In summary, the co-expression of GroEL/S from pGro7 in *V. natriegens* and *E. coli* had the most beneficial effect on soluble recombinant protein formation and hence on aminoacylase activity in the cell-free extract. This is in line with data from literature which also indicate that a chaperone often enhances solubility of the protein of interest, while others have no or even negative effects [3, 34–36].

### Expression in ***E. coli*** ArcticExpress

**Fig. 5:**
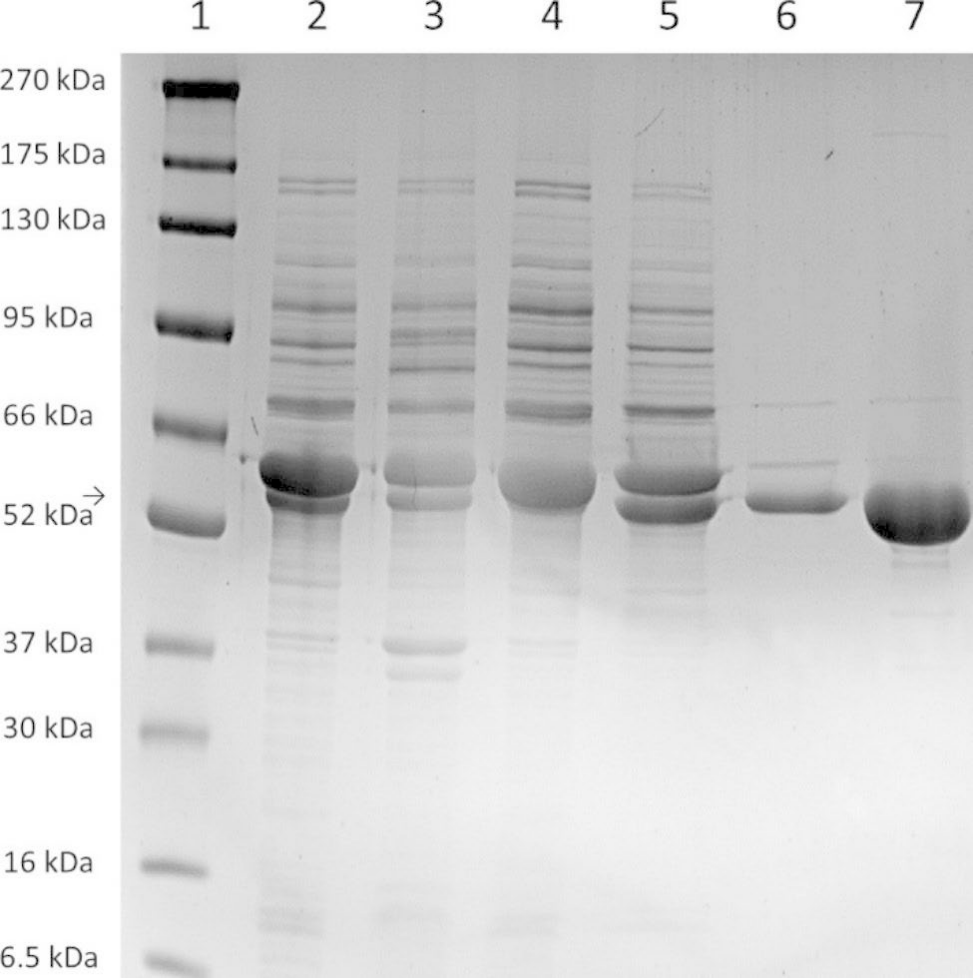
SDS-PAGE of MsAA NTag overexpression and Strep-tag purification from *E. coli* ArcticExpress (DE3). Lane 1: Protein marker (BlueEasy Prestained Protein Marker, Nippon Genetics); lane 2: cell-free extract; lane 3: insoluble fraction; lane 4: Flow-through; lane 5/6: wash fractions; lane 7: Elution of MsAA NTag.

Since in both *V. natriegens* Vmax and *E. coli* BL21 (DE3), GroEL/S had a positive effect on the production of soluble heterologous aminoacylase and lowering the temperature to 20 °C increased the yield of recombinant protein, we combined these findings by using another chaperone co-expressing strain, *E. coli* ArcticExpress (DE3), for production of MsAA. *E. coli* ArcticExpress carries a plasmid for expression of a cold-adapted chaperonin Cpn60/10, a homolog of GroEL/S isolated from *Oleispira antarctica*, a psychrophilic bacterium. This chaperonin has its temperature optimum in a range of 4 to 12 °C [33] allowing expression at a temperature of 12 °C. Aminoacylase activity and specific activity in the cell-free extract was measured as 139.1 (± 6.9) U/ml and 9.2 (± 0.5) U/mg, respectively. This were the highest activities observed among the strains used for MsAA overexpression and thus, *E. coli* ArcticExpress was used for production of MsAA for biochemical characterization. The enzyme was purified to homogeneity and specific activity of purified MsAA from *E. coli* ArcticExpress was 127.2 (± 4.8) U/mg. The SDS-PAGE analysis of cell extracts purification is shown in Fig. 5. The theoretical mass of purified MsAA containing the N-terminal Strep-tag was verified by MALDI-TOF analysis as 49.9 kDa (Additional file 1: Fig. S13). Since members of the M20 peptidase family can be both monomeric or multimeric enzymes, native PAGE was performed with MsAA and reference proteins. We found that the recombinant MsAA is a dimeric protein (Additional file 1: Fig. S14). By isoelectric focusing, it was shown that the enzyme has a pI of approximately 4.3 (Additional file 1: Fig. S15).

## Biochemical characterization of MsAA

### Effect of pH and temperature on activity and stability of recombinant MsAA

**Fig. 6:**
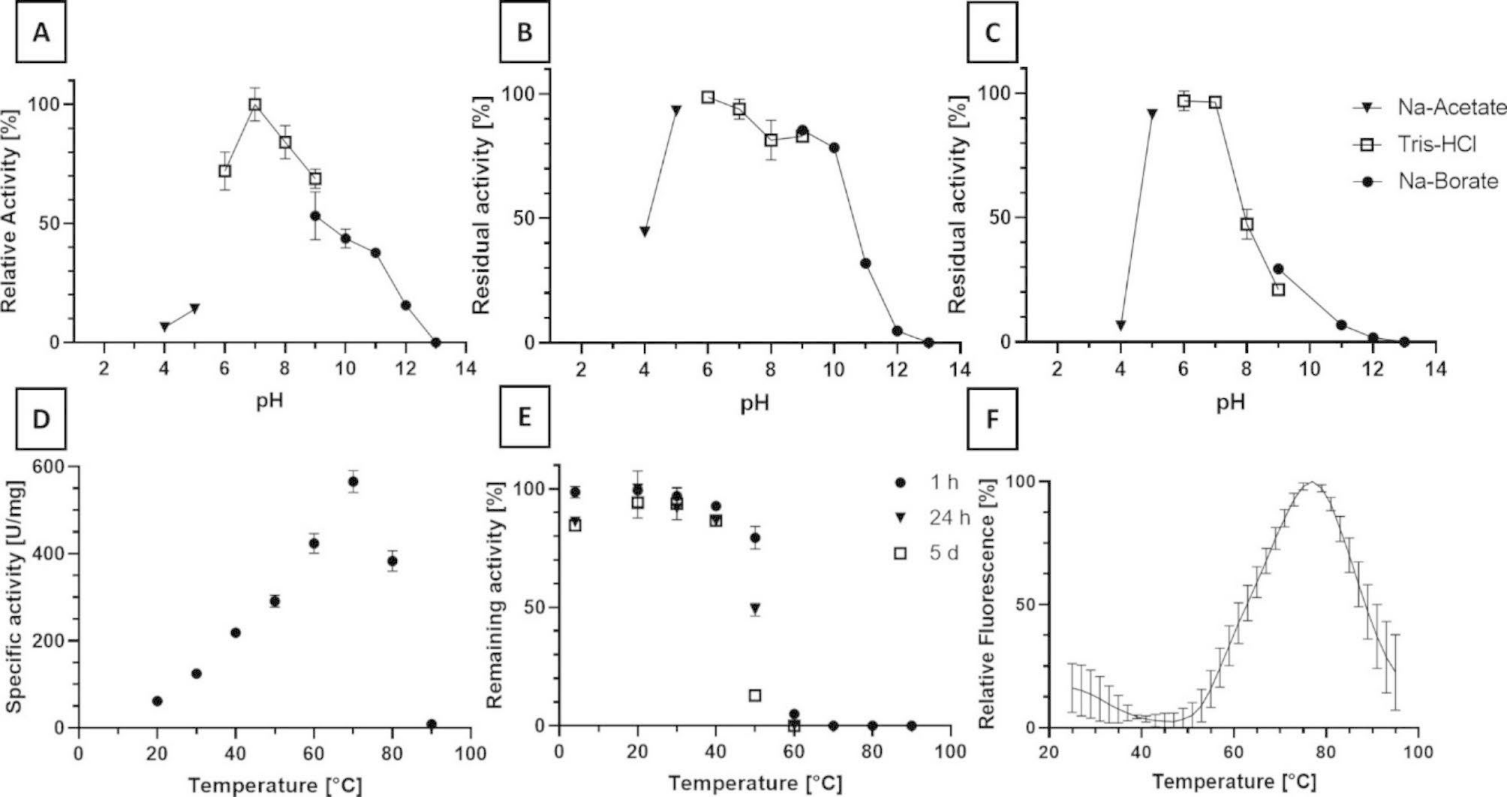
Biochemical characterization of MsAA **(A)** pH dependency of hydrolytic activity. Reaction conditions: 15 mM acetyl-alanine at 30 °C reaction temperature. The following buffers were used at 100 mM: Na-acetate for pH 4.0 and 5.0, Tris-HCl for pH 6.0–9.0, Na-borate for pH 9.0–13.0. **(B)** pH dependency of stability after 24 h and **(C)** after 5 d at 30 °C. Buffers and corresponding symbols used are identical to the pH activity optimum. Residual activity was determined with the standard assay conditions and indicated as percental values. **(D)** Temperature dependency on hydrolytic activity. Reaction conditions were 15 mM acetyl-alanine in 100 mM Tris-HCl pH 7.0 at various reaction temperatures. **(E)** Thermal stability after 1 h, 24 h and 5 days incubation at various temperatures in 100 mM Tris-HCl buffer pH 7.0. **(F)** Thermal shift assay. Thermal denaturation of MsAA was followed via fluorescence measurement of SYPRO Orange. All reactions were conducted in triplicates

The purified recombinant aminoacylase MsAA was characterized regarding its properties in hydrolytic reactions. The optimal pH for the hydrolytic reaction was pH 7.0 with 127.2 (± 4.8) U/mg (Fig. 6(A)). Investigation of the pH-dependent stability revealed good stability at neutral pH values, as the enzyme was most stable at pH 6.0 and 7.0. Stability of the enzyme quickly decreased at pH values of 4.0 and 11.0 (Fig. 6(B and C)). After 24 h incubation, the enzyme was still relatively stable in alkaline buffers up to pH 10.0. After 5 days, however, 47.3% activity remained at pH 8.0. Thus, the recombinant aminoacylase is best stored at pH 7.0 and storage buffer was 100 mM Tris-HCl pH 7.0 150 mM NaCl 1 mM ZnCl_2_.

With the optimal hydrolytic reaction temperature being 70 °C with 565.8 (± 25.3) U/mg, the enzyme is more active at higher temperatures (Fig. 6(D)). However, the stability starts to decrease at 50 °C. At 40 °C, the enzyme is stable even for five days (Fig. 6(E)). The thermal shift assay also revealed that thermal denaturation starts at approximately 50 °C (Fig. 6(F)). Considering its stability, reaction temperatures for the biocatalytic production of acyl-amino acids should not exceed 40 °C for a long time. The optimal pH for the condensation reaction might differ from the hydrolytic optimum. Still, the enzyme is stable at rather neutral or slightly acidic pH values of 6.0–7.0.

### Effect on metal ions and chelating agent EDTA on hydrolytic activity

We investigated the metal ion dependency by incubation with various divalent metal cations on MsAA. When the enzyme was purified without the addition of metal ions in the purification buffers, activity was 51% (65.2 ± 0.6 U/mg) of the activity with zinc added during purification. Addition of 1 mM EDTA to this enzyme preparation decreased its activity from 51 to 36% (45.7 ± 3.6 U/mg). When 1 mM EDTA was added to the enzyme saturated with 1 mM ZnCl_2_, activity barely decreased. The results suggest that the zinc ions bound to the enzyme can hardly be removed via complexation using the chelating agent EDTA.

The addition of MgSO_4_, MnCl_2_, CaCl_2_, or CoCl_2_ at a concentration of 1 mM to the enzyme purified without the addition of metal ions had no significant influence on hydrolytic activity, while 1 mM of NiSO_4_, FeSO_4_, and CuSO_4_ lowered the specific activity to 42% (54.4 ± 5.5 U/mg), 41% (52.9 ± 1.1 U/mg), and 40% (51.2 ± 0.2 U/mg), respectively. The addition of 1 mM ZnCl_2_ increased the activity to 84% (106.8 ± 8.6 U/mg) referring to activity when MsAA was purified with 1 mM zinc ions in each buffer. The results clearly indicate that MsAA is a zinc-dependent enzyme, as it is typical for members or homologues of the M20 peptidase family. Incubating the enzyme at ZnCl_2_ concentrations from 1 nM to 5 mM revealed that a minimal concentration of 10 µM ZnCl_2_ is needed in the enzyme storage buffer for full activity of MsAA. The results are summarized in Table 1.

**Table 1:** Effect on metal ions and chelating agent EDTA on hydrolytic activity. The enzyme purified without divalent cations added served as the control. The divalent metal salts or EDTA were added to this sample. The highest activity was observed when 1 mM ZnCl_2_ was added in all purification buffers and was set to 100%

Metal ion or chelating agent	Relative activity [%]	Specific activity [U/mg]
Control	51	65.2 (± 0.6)
1 mM EDTA	36	45.7 (± 3.6)
Purified with 1 mM ZnCl_2_	100	127.2 (± 0.6)
Purified with 1 mM ZnCl_2_, 1 mM EDTA added	96	122.7 (± 1.6)
1 mM ZnCl_2_	84	106.8 (± 8.6)
0.5 mM ZnCl_2_	81	103.5 (± 6.6)
0.1 mM ZnCl_2_	84	107.3 (± 5.8)
0.05 mM ZnCl_2_	85	107.9 (± 6.2)
0.01 mM ZnCl_2_	86	109.7 (± 4.0)
0.001 mM ZnCl_2_	65	82.3 (± 7.0)
1 mM MgSO_4_	47	60.3 (± 4.2)
1 mM MnCl_2_	48	60.9 (± 4.4)
1 mM CaCl_2_	50	63.8 (± 2.1)
1 mM CoCl_2_	52	66.0 (± 8.7)
1 mM NiSO_4_	43	54.4 (± 5.5)
1 mM FeSO_4_	42	52.9 (± 1.1)
1 mM CuSO_4_	40	51.2 (± 0.1)

### Hydrolytic substrate specificity

The hydrolytic substrate specificity was determined against various acyl-amino acids. The enzyme generally prefers amino acids acylated with acetic acid compared to longer or bulkier acyl chains (Table 2). In the case of alanine, valine, isoleucine and leucine, the ratio of hydrolytic activity against lauroyl-amino acids and the respective acetyl-amino acids is only 7.7%, 2.2%, 0.8% and 0.3%, respectively. In contrast, the activity against lauroyl-methionine is 39.0% of the activity against acetyl-methionine. Hence, lauroyl-methionine is accepted well by MsAA and hydrolytic activity against this substrate is comparable to other acetyl-amino acids. Comparing the substrate specificity of MsAA with its homologue SmAA from *S. mobaraensis*, similarities exist in that SmAA also prefers shorter acyl residues. Regarding the fatty acyl chain length, SmAA prefers octanoic acid [7]. However, we found in the case of glutamine, that acetyl-glutamine is strongly preferred over octanoyl-glutamine, which was barely hydrolyzed, and lauroyl- and palmitoyl-glutamine were not accepted at all by MsAA. Furthermore, MsAA is specific to substrates acylated at the alpha-amino position, as N_α_-acetyl-lysine was hydrolyzed with 18.1 (± 0.6) U/mg, whereas no activity against N_ε_-acetyl-lysine could be detected. The substrate specificity of MsAA is in approximate accordance with the substrate specificity of short acyl aminoacylase isolated from *M. smegmatis* ATCC 607 [19].

**Table 2:** Hydrolytic substrate specificity of MsAA. Substrates were used at 15 mM concentration in 100 mM Tris-HCl pH 7.0. Reaction temperature was elevated to 50 °C to ensure solubilization of all substrates

Substrate	Activity [U/mg]
Acetyl-alanine	265.9 (± 7.8)
Benzoyl-alanine	2.7 ± (0.1)
Lauroyl-alanine	20.6 (± 1.4)
Palmitoyl-alanine	20.7 (± 0.8)
Acetyl-methionine	140.9 (± 6.6)
Lauroyl-methionine	54.9 (± 0.7)
Acetyl-valine	326.1 (± 5,7)
Lauroyl-valine	7.1 (± 0.8)
Acetyl-isoleucine	284.2 (± 13.4)
Lauroyl-isoleucine	2.2 (± 0.3)
Acetyl-glutamine	44.6 (± 1.7)
Octanoyl-glutamine	0.8 (± 0.1)
Lauroyl-glutamine	0
Palmitoyl-glutamine	0
Acetyl-leucine	147.1 (± 6.8)
Lauroyl-leucine	0.5 (± 0.1)
Acetyl-threonine	63.5 (± 2.1)
Acetyl-aspartic acid	41.0 (± 0.6)
Lauroyl-aspartic acid	0
Acetyl-glycine	89.6 (± 1.8)
Lauroyl-glycine	2.7 (± 0.1)
Acetyl-glutamic acid	27.5 (± 1.4)
Acetyl-arginine	17.5 (± 0.6)
Acetyl-asparagine	20.3 (± 0.7)
Acetyl-cysteine	39.4 (± 2.5)
Lauroyl-cysteine	0
Acetyl-phenylalanine	3.4 (± 0.2)
Lauroyl-phenylalanine	0
Acetyl-tyrosine	1.2 (± 0.1)
Lauroyl-tyrosine	0
N_α_-acetyl-lysine	18.1 (± 0.6)
N_ε_-acetyl-lysine	0
Acetyl-tryptophane	0
Lauroyl-tryptophane	0
Acetyl-proline	0

### Biocatalytic synthesis of lauroyl-amino acids in aqueous media

The hydrolytic substrate specificity of MsAA revealed that the aminoacylase strongly favors acyl-amino acids with short-chain acyl residues, while most lauroyl-amino acids were barely accepted as substrates under the chosen conditions. An exception was observed for the hydrolysis of lauroyl-methionine, which was hydrolyzed with almost half of the activity compared to hydrolysis of acetyl-methionine. To evaluate the synthetic potential of MsAA for lauroyl-amino acids, an excess of amino acids and lauric acid was deployed in an aqueous reaction system. Initial reaction conditions were chosen according to the results obtained from the hydrolytic reactions, namely L-amino acid and lauric acid in equimolar concentration at 100 mM in 100 mM Tris-HCl pH 7.0 at 40 °C for 3 days in triplicates. The reaction mixtures were analyzed with HPLC-UV/-ELSD and product concentrations were measured with the respective lauroyl-amino acid standard. Among all proteinogenic amino acids, acylation was only observed for alanine, isoleucine, leucine, methionine, phenylalanine, and valine. The measured product concentrations were 1.1, 5.8, 5.1, 7.4, 0.1, and 4.5 mM, respectively. Hence, a bias for smaller, hydrophobic amino acids can be observed for the synthesis reaction. In accordance with the hydrolytic substrate specificity, lauroyl-methionine was found to be the preferred substrate in the synthesis reaction as well. To verify the identity of the synthesized molecule, the reaction mixture was analyzed with LC-MS, which confirmed the production of lauroyl-methionine (Additional File 1: Fig. S16).

## Conclusions

In this work, a chaperone co-expression approach is described for the functional expression of a novel mycobacterial aminoacylase that is prone to inclusion body formation in *E. coli* and *V. natriegens*. We showed that *V. natriegens* was advantageous compared to *E. coli* when IPTG induction was used, as *V. natriegens* Vmax™ readily produced soluble recombinant aminoacylase. *V. natriegens* was superior to *E. coli* for short expression times of 4 h. We described chaperone co-expression in *V. natriegens* for the first time and showed that it can be used to optimize heterologous expression. In both *V. natriegens* and *E. coli*, co-expression of GroEL/S had the most pronounced influence on MsAA activity in the cell-free extract. However, *E. coli* BL21 (DE3) could only produce soluble MsAA by lactose autoinduction. Furthermore, we found that the use of *E. coli* ArcticExpress, which expresses Cpn60/10, combined with low cultivation temperatures, was best for expression of MsAA.

It has been described that the genetic elements of the Takara chaperone plasmid set, the p15A origin of replication and the *araBAD* promoter, function in *V. natriegens* [28]. We confirmed that the chaperone plasmids can be introduced to *V. natriegens* and that co-expression of the chaperones can influence the solubility of the protein of interest and hence activity obtained from recombinant expression. Since pGTf2 and pGKJE8 use the tetracycline inducible promoter *Pzt-1*, regulated by TetR, these genetic elements also seem to function in *V. natriegens*.

From all tested hosts, specific activity of purified MsAA was similar. We biochemically characterized the aminoacylase based on hydrolytic activities. Hydrolytic substrate specificity indicated favoring of short-chain acyl residues, except for lauroyl-methionine. Lastly, we demonstrated that the enzyme is suitable for the biocatalytic synthesis of N-lauroyl-L-methionine from lauric acid and methionine in an aqueous system. The enzyme is thus highly interesting for further investigation and biocatalytic process intensification to optimize production of acyl-amino acids.

## Materials and methods

### Chemicals and reagents

Amino acids, cultivation media components, Tris (tris(hydroxymethyl)aminomethane), metal salts and solvents were from Carl Roth (Germany), acetyl amino acids were obtained from Sigma-Aldrich. Reagents for molecular biology were from Thermo Fisher Scientific (USA). DNA oligonucleotide synthesis and DNA sequencing were performed by Eurofins Genomics (Germany). Strep-Tactin columns were from IBA (Germany). Products for isoelectric focusing and native PAGE, as well as lysozyme were purchased from SERVA (Germany). Other chemicals were purchased from Sigma-Aldrich (USA). The EZ Nin reagent for amino acid quantification was from Biochrom (UK).

### Bacterial strains and plasmids

The microbial strains and plasmids used in this study are listed and described in Table 3. For cloning and plasmid maintenance, *E. coli* DH5α was used. For expression experiments, various *E. coli* strains were used. *E. coli* BL21 was used for most expression experiments. As an alternative strain deficient of the *lac*-operon, *E. coli* Tuner (DE3) was used. Furthermore, *E. coli* ArcticExpress was used for expression at low temperatures. As an alternative expression host, *Vibrio natriegens* Vmax™ was investigated. The co-expression with molecular chaperones was realized by co-transforming the strains with the chaperone plasmid set. Expression of MsAA was realized with pET28a MsAA NTag/CTag/noTag.

**Table 3:** Strains and plasmids used in this study

Strain/Plasmid	Genotype/Description	Source
*E. coli* DH5α	F^–^ φ80*lac*ZΔM15 Δ(*lac*ZYA-*arg*F)U169 *rec*A1 *end*A1 *hsd*R17(r_K_^–^, m_K_^+^) *pho*A *sup*E44 λ^–^*thi*-1 *gyr*A96 *rel*A1	Thermo Fisher Scientific (USA)
*E. coli* BL21 (DE3)	F^–^*ompT gal dcm lon hsdS*_*B*_(*r*_*B*_^–^*m*_*B*_^–^) λ(DE3 [*lacI lacUV5*-*T7p07 ind1 sam7 nin5*]) [*malB*^+^]_K−12_(λ^S^)	Thermo Fisher Scientific (USA)
*E. coli* Tuner (DE3)	F^–^*ompT hsdS*_B_ (r_B_^–^ m_B_^–^) *gal dcm lacY1*(DE3)	Novagen®, Merck (Germany)
*E. coli* ArcticExpress	F^–^*ompT hsdS* (r_B_^–^m_B_^–^) *dcm*^+^ Tet^r^*gal* λ(DE3) *endA* Hte [*cpn10 cpn60* Gent^r^]	Agilent Technologies (USA)
*Vibrio natriegens* Vmax™	*Vibrio natriegens* 14,048 dns::LacI-T7-RNAP	Telesis Bio (USA)
pET28a MsAA NTag/CTag/noTag	Derived from pET28a-eforRED[37], with coding sequence of MsAA with N- or C-terminal Strep-Tag, or without affinity tag	This study
pGKJE8	Arabinose-induced expression of *dnaK/J/grpE*; tetracycline-induced expression of *groEL/S*	Takara Bio Europe (France)
pKJE7	Arabinose-induced expression of *dnaK/J/grpE*	
pGTf2	Tetracycline-induced expression of *dnaK/J/grpE* and *tig* (Trigger factor)	
pTf16	Arabinose-induced expression of *tig*	
pGro7	Arabinose-induced expression of *groEL/S*	

### Cloning of the aminoacylase gene from ***Mycolicibacterium smegmatis*** MKD 8

The *msAA* gene was commercially ordered and synthesized by GeneArt (Thermo Fisher Scientific, USA). The gene was codon-optimized for *E. coli* with added sequences coding for additional StrepII-tag (WSHPQFEK) sequences at both ends, each connected with a linker (SG). The gene was amplified with primers carrying BsaI overhangs for Golden Gate cloning into pET28-eforRED, which derived from pET28a(+) (Novagen) [37]. Depending on the primer sequence, the aminoacylase gene could thus be cloned either with an N-terminal or a C-terminal Strep-tag, or without any affinity tag (Primers P1 & P4, P2 & P3 and P2 & P4, respectively; sequences for protein, gene and primers in Additional file 1: Table S1).

### Database searches and sequence analysis

Database searches were performed by using the BLASTp service from NCBI (National Center for Biotechnology Information) (https://blast.ncbi.nlm.nih.gov/) [38]. The alignment of multiple homologous sequences was conducted using the T-Coffee algorithm from EMBL-EBI (https://www.ebi.ac.uk/Tools/msa/tcoffee/) [39]. The alignment was displayed using ESPript 3.0 (https://espript.ibcp.fr/) [40]. ColabFold was used to predict a putative three-dimensional protein structure from the protein sequence of MsAA, based on the AlphaFold2 algorithm [41] and was used to add secondary structure elements to sequence alignments. For analysis of metal-binding sites and generation of a protein structure with added zinc ions, the MIB: Metal Ion-Binding Site Prediction and Docking Server was used [42].

### Generation of chemically competent bacterial cells and transformation

For the preparation of chemically competent *E. coli* according to [43] with TMF buffer (100 mM CaCl_2_, 50 mM RbCl, 40 mM MnCl_2_) for final storage of the competent cells. The generation of chemically competent *V. natriegens* Vmax™ and subsequent transformation was conducted according to [28].

### Production of recombinant aminoacylase in ***E. coli*** or ***V. natriegens*** and purification

Various media and protocols were used for heterologous aminoacylase expression depending on the organisms and strains. Furthermore, the T7-based expression was either induced by addition of IPTG or by autoinduction with lactose. For *E. coli*, precultures were grown in 10 ml LB medium overnight at 37 °C supplemented with the necessary antibiotics. For *E. coli* BL21 (DE3) and Tuner (DE3), 100 ml Terrific Broth medium was used (TB; 2% tryptone from casein, 2.4% yeast extract, 25 mM NaH_2_PO_4_, 25 mM KH_2_PO_4_, 50 mM NH_4_Cl, 2 mM MgSO_4_, 5 mM Na_2_SO_4_, 0.5% glycerol (v/v) and 0.05% glucose). The expression cultures were inoculated with an OD_600_ of 0.05. The cultures were induced with 1 mM IPTG once an OD_600_ of 0.5 was reached. The autoinduction with lactose was realized by adding 0.2% lactose to the medium (TB-AIM). The cells were cultivated for 4 h upon induction with IPTG, while the bacteria were cultivated for 24 h after inoculation when using autoinduction. Temperatures during the expression were either 20 or 30 °C. *E. coli* ArcticExpress (DE3) was cultivated for 24 h at 12 °C in TB autoinduction medium, with prior growth for 6 h at 30 °C. Furthermore, no antibiotics were added in the expression cultures of *E. coli* ArcticExpress (DE3). For co-expression of chaperones with *E. coli*, 0.5 mg/ml arabinose and/or 5 ng/ml tetracycline were added.

For *V. natriegens*, additional v2 salts (204 mM NaCl, 4.2 mM KCl, 23.4 mM MgCl_2_) were added to the media. Pre-cultures were grown overnight at 37 °C in 10 ml LBv2 medium. For expression in *V. natriegens* Vmax™, BHIv2 medium (37 g/l Brain-Heart-Infusion Broth, Carl Roth, Germany; with v2 salts) or TBv2 medium was used. Like for *E. coli*, the cultures were inoculated to an OD_600_ of 0.05, induced with 1 mM IPTG at OD_600_ of 0.5 and then cultured for 4 h at 30 °C (if not stated otherwise). For co-expression of chaperones, 4 mg/ml arabinose and/or 10 ng/ml tetracycline were added.

The cells were harvested by centrifugation at 3000 g and 4 °C for 40 min. To each cell pellet, harvested from 50 ml expression culture, 10 ml of lysis buffer was added (100 mM Tris-HCl pH 7.0 supplemented with 0.1% Triton X-100, 1 mM ZnCl_2_, 0.3 mg ml^− 1^ lysozyme and 150 mM NaCl) and the cells were disrupted by sonication (sonotrode MS 73, Bandelin, Germany). After lysis, the samples were centrifuged at 16,000 g and 4 °C for 60 min. The supernatant containing soluble protein was separated and the insoluble pellet was resuspended in 5 ml of 5 M urea for SDS-PAGE analysis.

Strep-tag affinity purification of recombinant MsAA NTag from the soluble fraction was conducted using a 5 ml Strep-Tactin® SuperFlow® high capacity cartridge according to manufacturer’s instructions. The buffer system consisted of 100 mM Tris-HCl pH 7.0, 1 mM ZnCl_2_ and 150 mM NaCl with 2.5 mM desthiobiotin for the elution buffer. The fractions containing sufficient enzyme concentrations were pooled and rebuffered to the same buffer without desthiobiotin in Vivaspin™ 6 concentrators (10000 MWCO; Sartorius, Germany).

### Determination of protein concentration, purity, and molecular mass

Protein concentrations were determined with the method of Bradford [44] using the Roti®-Nanoquant reagent (Carl Roth). Bovine serum albumin (BSA) served as a standard. The SDS-polyacrylamide gel electrophoresis of proteins was performed according to Laemmli [45] by using 8–20% gradient gels and staining with Roti®Blue quick (Carl Roth). As a protein marker, FastGene® BlueEasy Protein Marker (Nippon Genetics) was used.

Native molecular weight was determined by performing blue native PAGE using reagents from SERVA (Germany). SERVAGel™ N4-16% gels were used for electrophoresis, conducted as described by manufacturer’s protocols. The isoelectric point of the purified, desalinated enzyme was determined by isoelectric focusing (IEF) using the Servalyt™Precotes™ gel and the IEF marker pH 3–10 by SERVA (Germany) according to manufacturer’s protocols. Desalination of the purified protein was conducted by buffer exchange to 10 mM Tris-HCl pH 7.0 using Spin Columns (3 kDa cut-off, VWR).

Matrix-assisted laser desorption ionization-time of flight mass spectrometry (MALDI-TOF/MS) analysis was used to determine the molecular mass of MsAA NTag. The device used for analysis was Axima Confidence (Shimadzu Europe, Duisburg, Germany), which was operated in linear positive mode with pulsed extraction optimized for the theoretical molecular weight. The mMass software was used for data analysis [46]. Protein solutions were diluted to 1 mg/ml in enzyme storage buffer. The samples were then diluted tenfold with α-Cyano-4-hydroxycinnamic acid (CHCA) (Sigma-Aldrich, USA). Per spot on the MALDI target plate, 2 µl of the sample were applied. Trypsinogen and BSA (Laserbio, France) were used as molecular weight standards.

### Aminoacylase activity assay

Activity of aminoacylases was assayed by quantification of released amino acids with a ninhydrin-based assay as previously described [47]. Briefly, 10 µl sample from amino acid solutions or aminoacylase reactions were mixed with 100 µl of EZ Nin:DMSO reagent, heated for 10 min at 99 °C and diluted with 100 mM Na-borate buffer pH 10.0 for measurement. In general, 200 µl reactions consisted of 190 µl substrate solution and 10 µl enzyme solution. For standard hydrolysis activity measurement, reaction with 15 mM N-acetyl-L-alanine in 100 mM Tris-HCl buffer pH 7.0, 50 µM ZnCl_2_, were performed at 30 °C for 5 min. At 1 min sampling intervals, 10 µl samples were withdrawn for the ninhydrin reaction. One unit of MsAA was defined as the amount of enzyme that hydrolyzes one µmol of N-acetyl-L-alanine per minute under the given conditions.

### Biochemical properties of aminoacylase

For the determination of the pH dependency of the hydrolytic reaction rate, reactions were carried out in the following buffers: Na-acetate for pH 4.0 and 5.0, Tris-HCl for pH 6.0–9.0 and Na-borate for pH 9.0–13.0. As substrate solution, 15 mM N-acetyl-L-alanine was prepared in 100 mM of the respective buffer and adjusted to respective pH at 30 °C. The purified enzyme concentrations in the assay were 10–100 µg/ml.

For investigating pH stability, buffers used were Na-acetate for pH 4.0 and 5.0, Tris-HCl for pH 6.0–9.0 and Na-borate for pH 9.0–13.0, all at concentrations of 100 mM. The enzyme solution was diluted 10-fold with the incubation buffers to a concentration of 200 µg/ml and incubated at 30 °C. From the incubation solutions, 10 µl was withdrawn for standard aminoacylase activity measurements after 24 h and 3 days. The final enzyme concentration was 10 µg/ml.

The optimal temperature for the hydrolysis was determined in a range of 20–90 °C with standard aminoacylase activity. The pH of the solutions was set at the respective temperatures. For the assessment of temperature stability, purified MsAA was incubated at temperatures from 20 to 90 °C and residual activity was determined after 1 h, 24 h, 5 days and 7 days. As incubation buffers, 100 mM Tris-HCl was set to pH 7.0 at the respective temperature. The purified enzyme was incubated at 380 µg/ml and 1 mM ZnCl_2_. Residual activity was determined with standard aminoacylase assay.

Recombinant MsAA was purified without any metal ions added to the buffers to investigate the effect of metal ions or chelating agents added to the purified enzyme. Various bivalent metal ions (CaCl_2_, CoCl_2_, CuCl_2_, FeSO_4_, MgCl_2_, MnCl_2_, NiCl_2_, ZnCl_2_) were added to a concentration of 1 mM to the enzyme solution and incubated for 1 h at room temperature before measuring standard aminoacylase activity. For ZnCl_2_, concentrations were set from 1 µM to 5 mM. The influence of 1 mM ethylenediaminetetraacetic acid (EDTA) as a chelating agent of bivalent ions was also investigated and residual activity was measured after 1 h incubation at room temperature.

The substrate specificity was determined by hydrolysis of various substrates at 15 mM concentration in 100 mM Tris-HCl pH 7.0 at 50 °C and 50 µM ZnCl_2_. The reaction temperature was higher than the standard conditions to solubilize all substrates. The concentrations of purified enzyme were 5- 100 µg/ml.

### Determination of thermal denaturation

With the thermal shift assay, thermal denaturation of the investigated protein was accessed as previously described [48]. The fluorogenic dye Sypro orange was mixed with the protein and heated stepwise. When the protein is folded correctly, the dye does not bind efficiently on the hydrophilic surface. When hydrophobic stretches are exposed due to denaturation of the protein, binding of the dye leads to an increase in fluorescence. For the assay, 10 µl protein samples (> 0.1 mg/ml) are mixed with 5 µl 50x SYPRO Orange (Sigma Aldrich) and 20 µl 10 mM HEPES pH 8.0. As a positive control, 10 mg/ml lysozyme (Serva) was used. Measurement was done with qTower3G and qPCRsoft 4.0 (Analytik Jena) using the TAMRA Channel (λ_ex_ = 535 nm, λ_em_ = 580 nm). The heating program was 25 to 95 °C with steps of 2 °C, 120 s hold time per temperature and a heating speed of 4.4 °C/s.

### Chemical synthesis of N-acyl-amino acids

*N*-lauroyl-, *N*-palmitoyl- and other *N-*acyl-amino acids were synthesized by the Schotten-Baumann-reaction following the protocols of Takehara [49]. Amino acids (70 mmol) and NaOH (1.40 g, 70 mmol, 1.0 eq) were dissolved in 49 ml H_2_O and 35 ml acetone. With glutamic or aspartic acid as substrates, 2.80 g NaOH (140 mmol, 2.0 eq.) were used for the initial dissolution in the water/acetone mixture. To the cooled mixtures (4 °C, 2000 rpm) 4.2 g (105 mmol, 1.5 eq) NaOH in 15 ml water and acyl chloride (84 mmol, 1.1 eq.) were added dropwise over a period of 30 min. The reactions were run for a total time of 4 h and allowed to warm to room temperature. Acidification with 12 N hydrochloric acid led to the precipitation of *N*-acyl-amino acids. The crude products were separated by filtration, carefully washed with water (40 ml) and washed twice with 100 ml of petroleum ether (bp = 40–60 °C). Remaining solvent was removed *in vacuo* and the white products were dried by lyophilization afterwards. The products were analyzed by HPLC and LC-MS. Purity data and HPLC retention times of the *N*-acyl-amino acids are provided in Additional file 1: Table S2.

### Biocatalytic synthesis of lauroyl-amino acids

Initial biocatalytic synthesis of lauroyl-amino acids was investigated from 100 mM L-amino acid and 100 mM lauric acid in 100 mM Tris-HCl pH 7.0 at 40 °C for 72 h at a reaction volume of 0.5 ml without agitation. Reactions were started by adding 10 µg of MsAA (1.3 U, according to standard activity assay). For analysis, a 100 µl sample was withdrawn and immediately mixed with 100 µl of a mixture of 80% acetonitrile and 20% water containing 0.1% trifluoroacetic acid (TFA).

### HPLC-UV/ELSD analysis

Analysis of biocatalytic reactions to investigate the synthesis of lauroyl-amino acids and to quantify product concentration, an HPLC system (S5200 and S2100; Sykam, Germany) equipped with a ISAspher 100-5 C18 BDS column (C18, 5 μm, 4.0 * 250 mm; Isera, Germany) coupled with UV (UV Detector 2500 Sykam, Germany)- and evaporative light scattering detectors (ELSD; ZAM 4000, Schambeck SFD, Germany) was used. The column was heated to 40 °C and isocratic separation was performed with a flow rate of 1 ml/min and a solvent mixture of 80% acetonitrile and 20% water containing 0.1 TFA. Concentration of lauroyl-amino acids was calculated with a respective external standard, analyzed by both UV (210 nm) and ELSD. As values obtained by both detectors were in good accordance, only concentrations obtained from UV measurement are shown.

### HPLC-MS analysis

A Shimadzu Nexera XR system equipped with a Hitachi LaChrom II column (C18, 5 μm, 4.6 * 250 mm) and a Shimadzu LCMS-2020-mass spectrometer was used to verify the correct mass of the product N-lauroyl-methionine from biocatalytic synthesis. The column was operated at 40 °C with a flow of 1 ml/min and the analytes were separated by applying a gradient run starting from a mixture of 20% acetonitrile and 80% water containing 0.1% formic acid, going to 100% acetonitrile in 10 min. The concentration was held for 6 min before returning to 20% acetonitrile over 2 min.

## Electronic supplementary material

Below is the link to the electronic supplementary material.

Additional file 1: MsAA protein sequence, primer sequences, gel electrophoresis results, chemically synthesized acyl-amino acids, MS spectrum for lauroyl-methionine.

## Data Availability

The datasets supporting the conclusions of this article are included within the article and its additional files.
