# Supplementary material for: Chaperone assisted recombinant expression of a mycobacterial aminoacylase in Vibrio natriegens and Escherichia coli capable of N-lauroyl-L-amino acid synthesis
Source: Microb Cell Fact. 2023 Apr 21;22:77. doi: 10.1186/s12934-023-02079-1 (PMC10122368; doi:10.1186/s12934-023-02079-1)
Supplement: Supplementary file 1 — Additional file 1: MsAA protein sequence, primer sequences, gel electrophoresis results, chemically synthesized acyl-amino acids, MS spectrum for lauroyl-methionine. [file 12934_2023_2079_MOESM1_ESM.docx]

**Supplement for:**

**Chaperone assisted recombinant expression in *V. natriegens* and *E. coli* and characterization of a mycobacterial aminoacylase capable of N-lauroyl-L-methionine synthesis**

Protein sequence of MsAA NTag (N-terminal Strep-tag and linker underlined):

MWSHPQFEKSGMVRMVTVTVSAASADEVVDLVSALIRFDTSNTGDPATTKGEAECAHWVAQQLEEVGYETEYVESGAPGRGNVFARLRGADPSRGALMVHGHLDVVPAEPADWSVHPFSGAVKDGYVWGRGAVDMKDMVGMTLAVARHFKRAGIVPPRDLVFAFVADEEHGGTYGADWLVNNRPDLFEGVTEAIGEVGGFSLTVPRKDGGERRLYLIETAEKGLSWMRLTARGRAGHGSMVHDDNAVTAIAGAVDRLGRHEFPLVLSPAVEEFLTAVAEETGYTFDPNSPDLEGTIAKLGGVARIVSATLRDTANPTMLKAGYKANVIPAVAEAMIDCRVLPGRKEAFEREVDELIGPDVTRSWERDLPSYETSFDGDLVDAMNASVLTLDPEARIVPYMLSAGTDAKSFQRLGIRCFGFAPLRLPPDLDFAALFHGVDERVPVDALQFGAGVLEHFLQNC

Codon-optimized sequence with N- and C-terminal strep-tag shown in bold letters (as ordered from GeneArt, Thermo Fisher)

**ATGTGGTCACATCCGCAGTTTGAAAAAAGCGGTATG**GTTCGTATGGTTACCGTTACCGTGAGCGCAGCAAGCGCAGATGAAGTTGTTGATCTGGTTAGCGCACTGATTCGTTTTGATACCAGCAATACCGGTGATCCGGCAACCACCAAAGGTGAAGCAGAATGTGCCCATTGGGTTGCACAGCAGCTGGAAGAGGTTGGTTATGAAACCGAATATGTTGAAAGCGGTGCACCTGGTCGTGGTAATGTTTTTGCACGTCTGCGTGGTGCAGATCCGAGCCGTGGTGCACTGATGGTTCATGGTCATCTGGATGTTGTTCCGGCAGAACCGGCAGATTGGAGCGTTCATCCGTTTAGCGGTGCAGTTAAAGATGGTTATGTTTGGGGTCGTGGTGCCGTTGATATGAAAGATATGGTTGGTATGACCCTGGCAGTTGCACGTCATTTTAAACGTGCAGGTATTGTTCCGCCTCGTGATCTGGTGTTTGCATTTGTTGCCGATGAAGAACATGGTGGCACCTATGGTGCCGATTGGCTGGTTAATAATCGTCCGGACCTGTTTGAAGGTGTTACCGAAGCAATTGGTGAAGTTGGTGGTTTTAGCCTGACCGTTCCGCGTAAAGATGGCGGTGAACGTCGTCTGTATCTGATTGAAACCGCAGAAAAAGGTCTGAGCTGGATGCGTCTGACCGCACGTGGTCGTGCAGGTCATGGTAGCATGGTGCATGATGATAATGCAGTTACCGCAATTGCCGGTGCGGTTGATCGTCTGGGTCGTCATGAATTTCCGCTGGTTCTGAGTCCGGCAGTTGAAGAATTTCTGACAGCAGTTGCAGAAGAAACCGGTTATACCTTTGATCCGAATAGTCCGGATCTGGAAGGCACCATTGCAAAACTTGGTGGTGTTGCACGTATTGTTAGCGCAACCCTGCGTGATACCGCAAATCCGACCATGCTGAAAGCAGGTTATAAAGCAAATGTGATTCCGGCAGTGGCAGAAGCAATGATTGATTGTCGTGTTCTGCCTGGTCGTAAAGAAGCATTTGAACGCGAAGTTGATGAACTGATTGGTCCGGATGTTACCCGTAGCTGGGAACGTGATCTGCCGAGCTATGAAACCAGCTTTGATGGCGATCTGGTTGATGCAATGAATGCAAGCGTTCTGACCCTGGATCCGGAAGCACGCATTGTTCCGTATATGCTGAGCGCAGGCACCGATGCAAAAAGCTTTCAGCGTCTGGGTATTCGTTGTTTTGGTTTTGCACCGCTGCGTCTGCCACCTGATCTGGATTTTGCAGCACTGTTTCATGGTGTGGATGAACGTGTTCCGGTTGATGCCCTGCAGTTTGGTGCCGGTGTTCTGGAACATTTCCTGCAGAATTGT**AGCGGTTGGAGCCATCCTCAGTTCGAGAAATGA**

Primer sequences for cloning:

Table S1: Primer sequences used for amplification and cloning of msAA.

BsaI-sites are highlighted in bold font.

| No. | Name | Sequence 5’->3’ |
| --- | --- | --- |
| P1 | NTag for: | **GGTCTC**CCATGTGGTCACATCCGCAGTTTGAAAAAAG |
| P2 | noTag for: | **GGTCTC**CCATGGTTCGTATGGTTACCGTTACCGTG |
| P3 | CTag rev: | **GGTCTC**TCTCATTTCTCGAACTGAGGATGGCTC |
| P4 | noTag rev: | **GGTCTC**TCTCAACAATTCTGCAGGAAATGTTCCAGAAC |

Table S2: Purity and HPLC-retention time of N-lauroyl-amino acids synthesized in this study.

Synthesis was conducted via Schotten-Baumann-method utilizing caproyl chloride (C8:0), lauroyl chloride (C12:0) and palmitoyl chloride (C16:0).

| **Amino acid** | ***Purity* [%]** | ***t_ret_* [min]** |
| --- | --- | --- |
| N-lauroyl-L-alanine | 94 | 13.3 |
| N-palmitoyl-L-alanine | 96 | 17.0 |
| N-lauroyl-L-arginine | 98 | 8.7 |
| N-lauroyl-L-aspartic acid | 89 | 8.5 |
| N-lauroyl-L-cysteine | 99 | 13.6 |
| N-lauroyl-L-glutamic acid | 97 | 11.2 |
| N-lauroyl-L-glycine | 99 | 12.8 |
| N-lauroyl-L-histidine | 60 | 8.6 |
| N-lauroyl-L-isoleucine | 98 | 14.3 |
| N-lauroyl-L-leucine | 99 | 14.3 |
| N-lauroyl-L-methionine | 99 | 13.8 |
| N-lauroyl-L-phenylalanine | 80 | 14.2 |
| N-lauroyl-L-serine | 94 | 18.6 |
| N_α_-lauroyl-L-threonine | 68 | 14.4 |
| N_α_-lauroyl-L-tryptophan | 98 | 13.8 |
| N-lauroyl-L-tyrosine | 97 | 14.1 |
| N-lauroyl-L-valine | 99 | 13.9 |
| N_α_-lauroyl-L-glutamine | 87 | 11.3 |
| N_α_-palmitoyl-L-glutamine | 82 | 14.6 |
| N_α_-caproyl-L-glutamine | 98 | 7.7 |


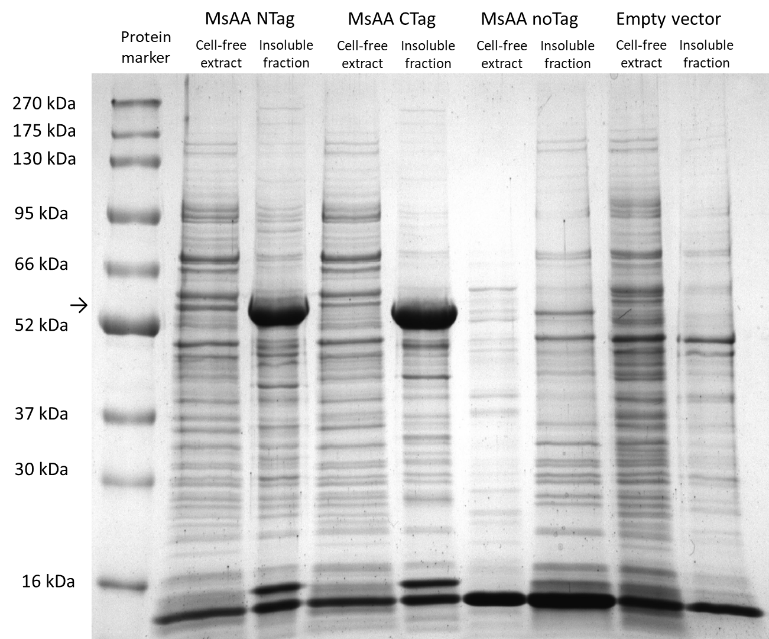


Figure S1: SDS-PAGE of expression of all MsAA tag-variants from V. natriegens Vmax™ at 37 °C.

Lane 1: protein marker (BlueEasy Prestained Protein Marker, Nippon Genetics); lane 2: cell-free extract with MsAA NTag; lane 3: insoluble fraction with MsAA NTag; lane 4: cell-free extract with MsAA CTag; lane 5: insoluble fraction with MsAA CTag; lane 6: cell-free extract with MsAA noTag; lane 7: insoluble fraction with MsAA noTag; lane 8: cell-free extract of empty vector control; lane 9: insoluble fraction of empty vector control


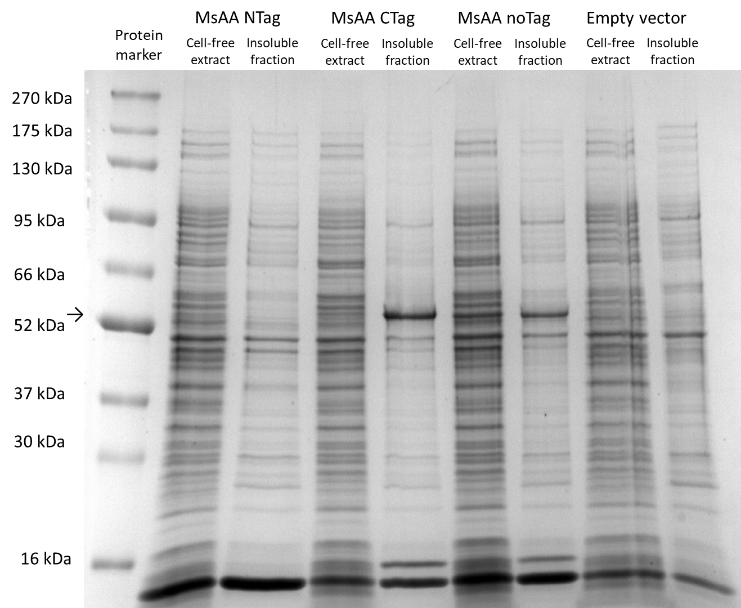


Figure S2: SDS-PAGE of expression of all MsAA tag-variants from V. natriegens Vmax™ at 20 °C.

Lane 1: protein marker (BlueEasy Prestained Protein Marker, Nippon Genetics); lane 2: cell-free extract with MsAA NTag; lane 3: insoluble fraction with MsAA NTag; lane 4: cell-free extract with MsAA CTag; lane 5: insoluble fraction with MsAA CTag; lane 6: cell-free extract with MsAA noTag; lane 7: insoluble fraction with MsAA noTag; lane 8: cell-free extract of empty vector control; lane 9: insoluble fraction of empty vector control


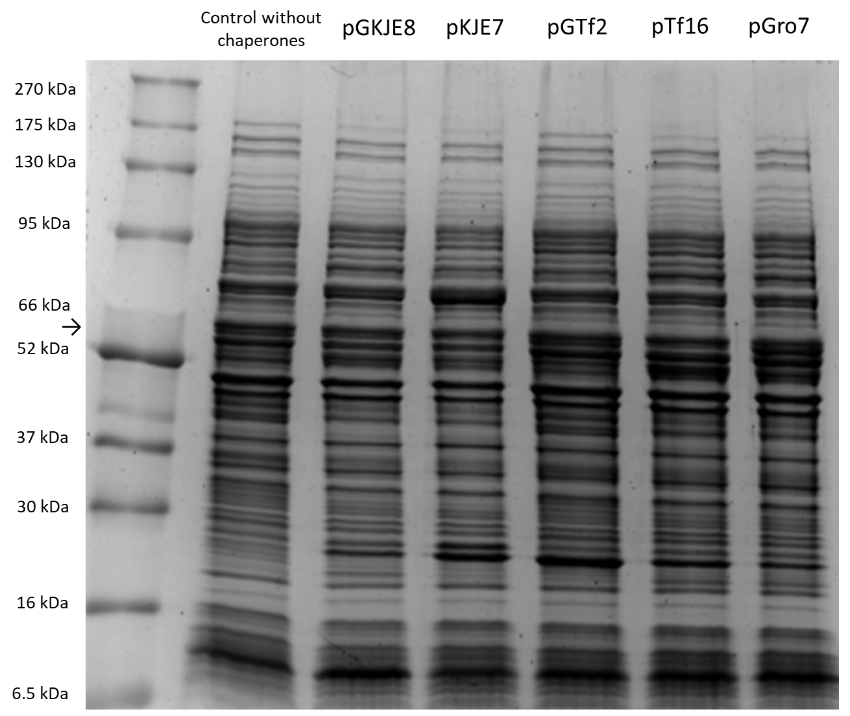


Figure S3: SDS-PAGE of soluble fractions from MsAA expression from V. natriegens Vmax™ with chaperone co-expression.

Lane 1: protein marker (BlueEasy Prestained Protein Marker, Nippon Genetics); lane 2: cell-free extract of empty vector control without chaperone co-expression; lane 3: cell-free extract of MsAA NTag expression without chaperone co-expression; lane 4: cell-free extract of MsAA NTag expression with GroEL/S and DnaK/J/GrpE chaperone co-expression from pGKJE8; lane 5: cell-free extract of MsAA NTag expression with DnaK/J/GrpE chaperone co-expression from pKJE7; lane 6: cell-free extract of MsAA NTag expression with GroEL/S and Trigger factor chaperone co-expression from pGTf2; lane 7: cell-free extract of MsAA NTag expression with Trigger factor chaperone co-expression from pTf16; lane 8: cell-free extract of MsAA NTag expression with GroEL/S chaperone co-expression from pGro7


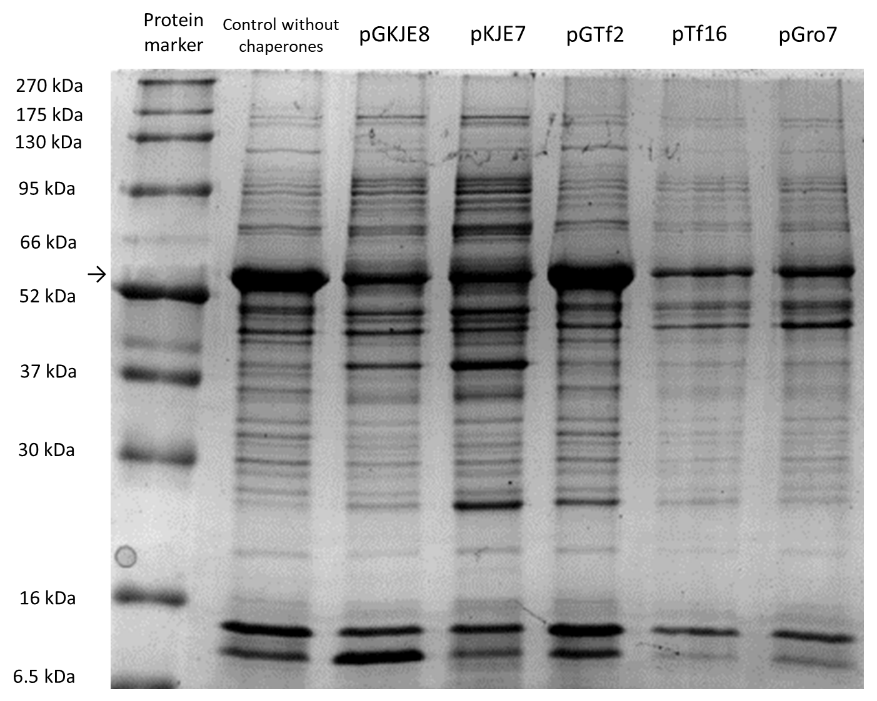


Figure S4: SDS-PAGE of insoluble fractions from MsAA from V. natriegens Vmax™ with chaperone co-expression.

Lane 1: protein marker (BlueEasy Prestained Protein Marker, Nippon Genetics); lane 2: insoluble fraction of empty vector control without chaperone co-expression; lane 3: insoluble fraction of MsAA NTag expression without chaperone co-expression; lane 4: insoluble fraction of MsAA NTag expression with GroEL/S and DnaK/J/GrpE chaperone co-expression from pGKJE8; lane 5: insoluble fraction of MsAA NTag expression with DnaK/J/GrpE chaperone co-expression from pKJE7; lane 6: insoluble fraction of MsAA NTag expression with GroEL/S and Trigger factor chaperone co-expression from pGTf2; lane 7: insoluble fraction of MsAA NTag expression with Trigger factor chaperone co-expression from pTf16; lane 8: insoluble fraction of MsAA NTag expression with GroEL/S chaperone co-expression from pGro7


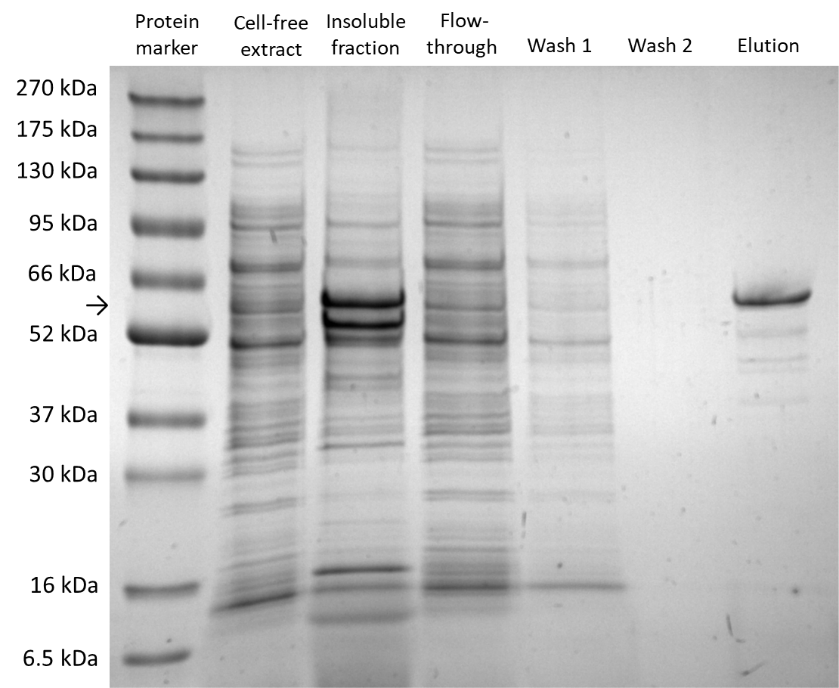


Figure S5: SDS-PAGE of MsAA NTag overexpression and purification from V. natriegens Vmax™ without chaperone co-expression.

Lane 1: Protein marker (BlueEasy Prestained Protein Marker, Nippon Genetics); lane 2: cell-free extract with MsAA NTag; lane 3: insoluble fraction with MsAA NTag; lane 4: Flow-through from purification; lane 5: first wash fraction; lane 6: second wash fraction; lane 7: Elution of MsAA NTag.


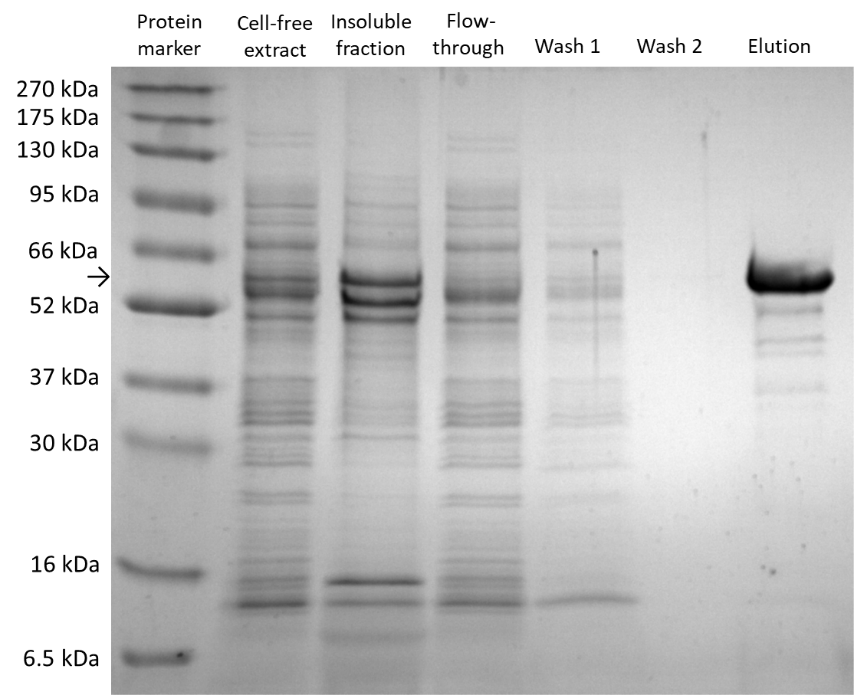


Figure S6: SDS-PAGE of MsAA NTag overexpression and purification from V. natriegens Vmax™ with GroEL/S co-expression.

Lane 1: Protein marker (BlueEasy Prestained Protein Marker, Nippon Genetics); lane 2: cell-free extract with MsAA NTag; lane 3: insoluble fraction with MsAA NTag; lane 4: Flow-through from purification; lane 5: first wash fraction; lane 6: second wash fraction; lane 7: Elution of MsAA NTag.


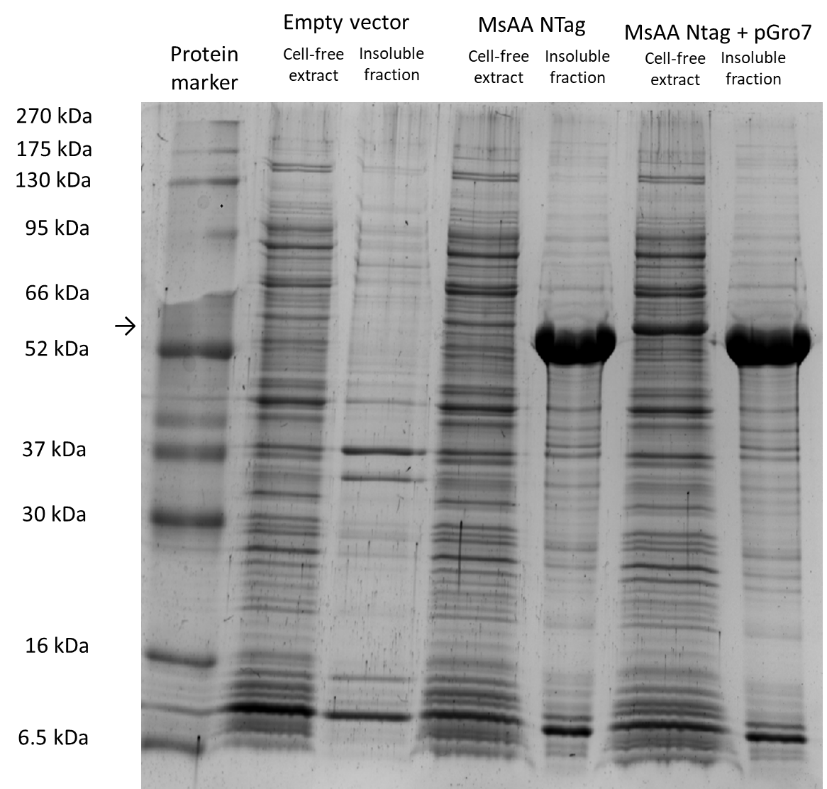


Figure S7: SDS-PAGE of IPTG-induced expression of MsAA NTag from E. coli BL21 (DE3) at 30 °C.

Lane 1: protein marker (BlueEasy Prestained Protein Marker, Nippon Genetics); lane 2: cell-free extract of empty vector control; lane 3: insoluble fraction of empty vector control; lane 4: cell-free extract with MsAA NTag; lane 5: insoluble fraction with MsAA NTag; lane 6: cell-free extract with MsAA NTag and GroEL/S co-expression from pGro7; lane 7: insoluble fraction with MsAA NTag and GroEL/S co-expression from pGro7.


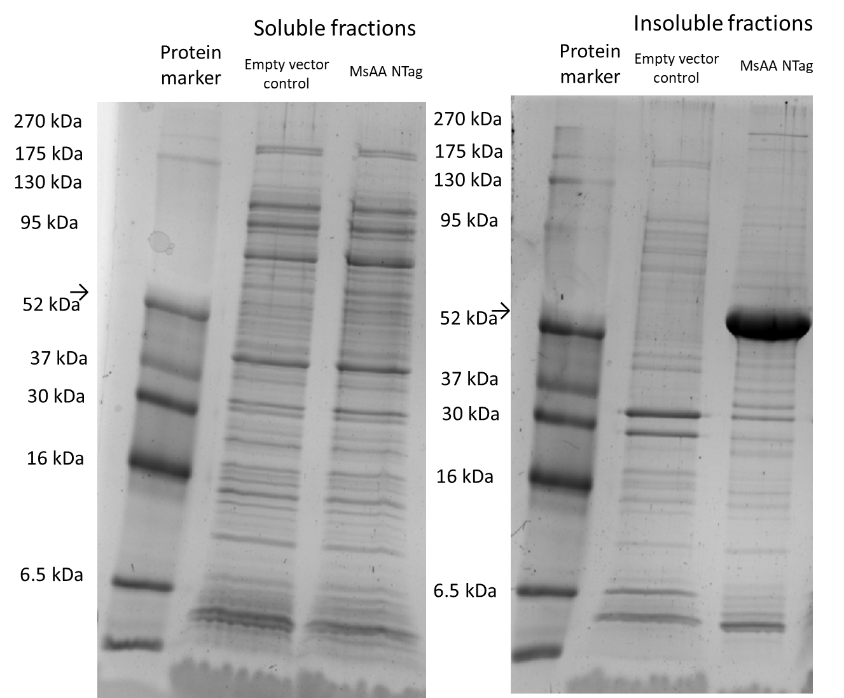


Figure S8: SDS-PAGE of IPTG-induced expression of MsAA NTag from E. coli Tuner™ (DE3) at 30 °C.

Lane 1: protein marker (BlueEasy Prestained Protein Marker, Nippon Genetics); lane 2: cell-free extract of empty vector control; lane 3: cell-free extract with MsAA NTag; lane 4: protein marker; lane 5: insoluble fraction of empty vector control; lane 6: insoluble fraction with MsAA NTag


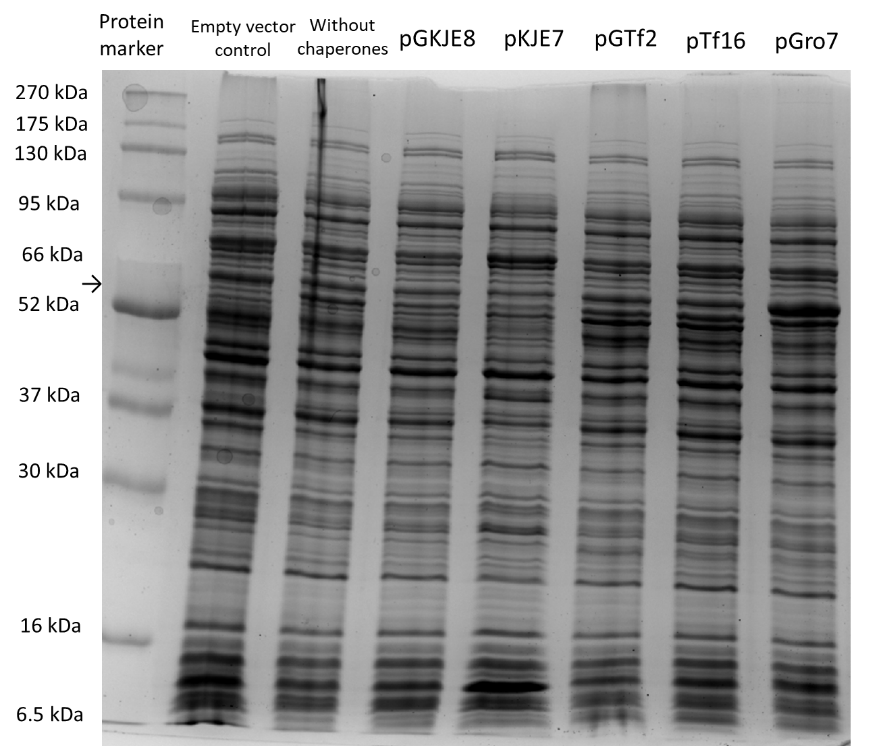


Figure S9: SDS-PAGE of soluble fractions from MsAA expression from E. coli BL21 (DE3) with chaperone co-expression

Lane 1: protein marker (BlueEasy Prestained Protein Marker, Nippon Genetics); lane 2: cell-free extract of empty vector control without chaperone co-expression; lane 3: cell-free extract of MsAA NTag expression without chaperone co-expression; lane 4: cell-free extract of MsAA NTag expression with GroEL/S and DnaK/J/GrpE chaperone co-expression from pGKJE8; lane 5: cell-free extract of MsAA NTag expression with DnaK/J/GrpE chaperone co-expression from pKJE7; lane 6: cell-free extract of MsAA NTag expression with GroEL/S and Trigger factor chaperone co-expression from pGTf2; lane 7: cell-free extract of MsAA NTag expression with Trigger factor chaperone co-expression from pTf16; lane 8: cell-free extract of MsAA NTag expression with GroEL/S chaperone co-expression from pGro7


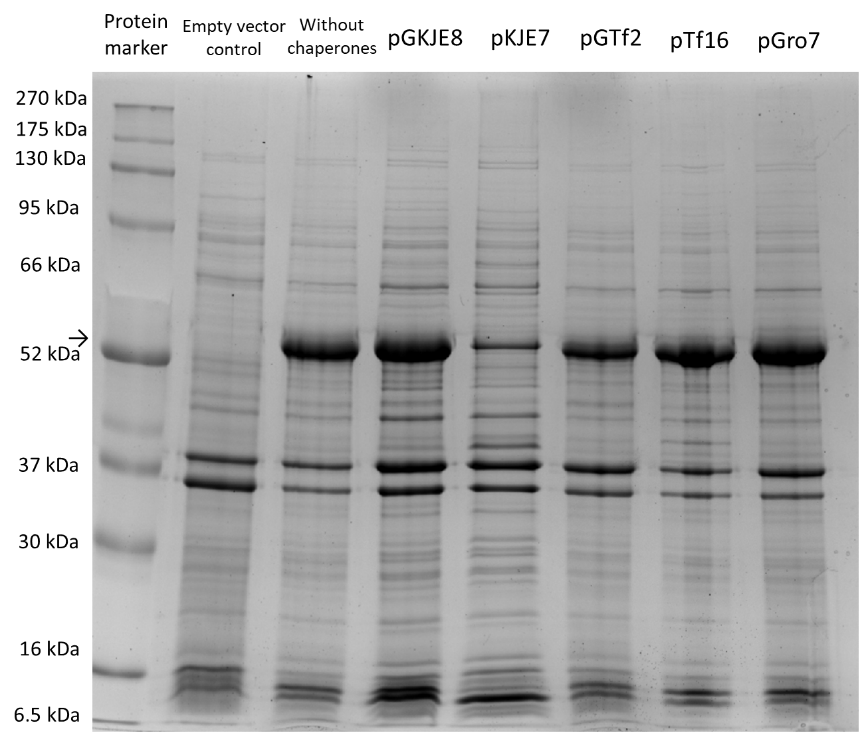


Figure S10: SDS-PAGE of insoluble fractions from MsAA expression from E. coli BL21 (DE3) with chaperone co-expression

Lane 1: protein marker (BlueEasy Prestained Protein Marker, Nippon Genetics); lane 2: insoluble fraction of empty vector control without chaperone co-expression; lane 3: insoluble fraction of MsAA NTag expression without chaperone co-expression; lane 4: insoluble fraction of MsAA NTag expression with GroEL/S and DnaK/J/GrpE chaperone co-expression from pGKJE8; lane 5: insoluble fraction of MsAA NTag expression with DnaK/J/GrpE chaperone co-expression from pKJE7; lane 6: insoluble fraction of MsAA NTag expression with GroEL/S and Trigger factor chaperone co-expression from pGTf2; lane 7: insoluble fraction of MsAA NTag expression with Trigger factor chaperone co-expression from pTf16; lane 8: insoluble fraction of MsAA NTag expression with GroEL/S chaperone co-expression from pGro7


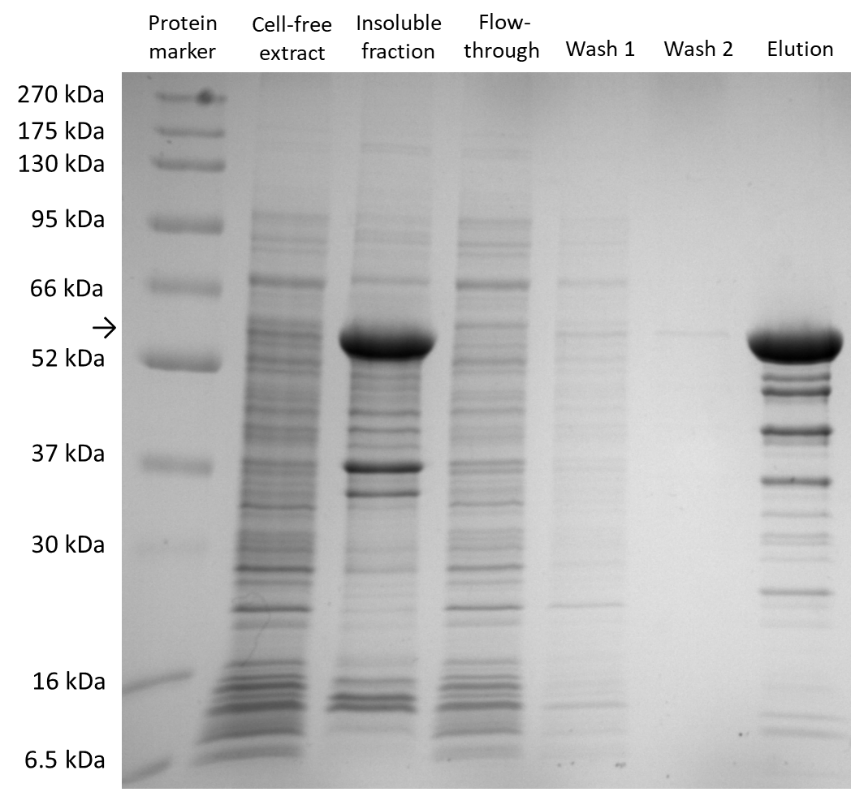


Figure S11: SDS-PAGE of MsAA NTag overexpression and purification from E. coli BL21 (DE3) without chaperone co-expression.

Lane 1: Protein marker (BlueEasy Prestained Protein Marker, Nippon Genetics); lane 2: cell-free extract with MsAA NTag; lane 3: insoluble fraction with MsAA NTag; lane 4: Flow-through from purification; lane 5: first wash fraction; lane 6: second wash fraction; lane 7: Elution of MsAA NTag.


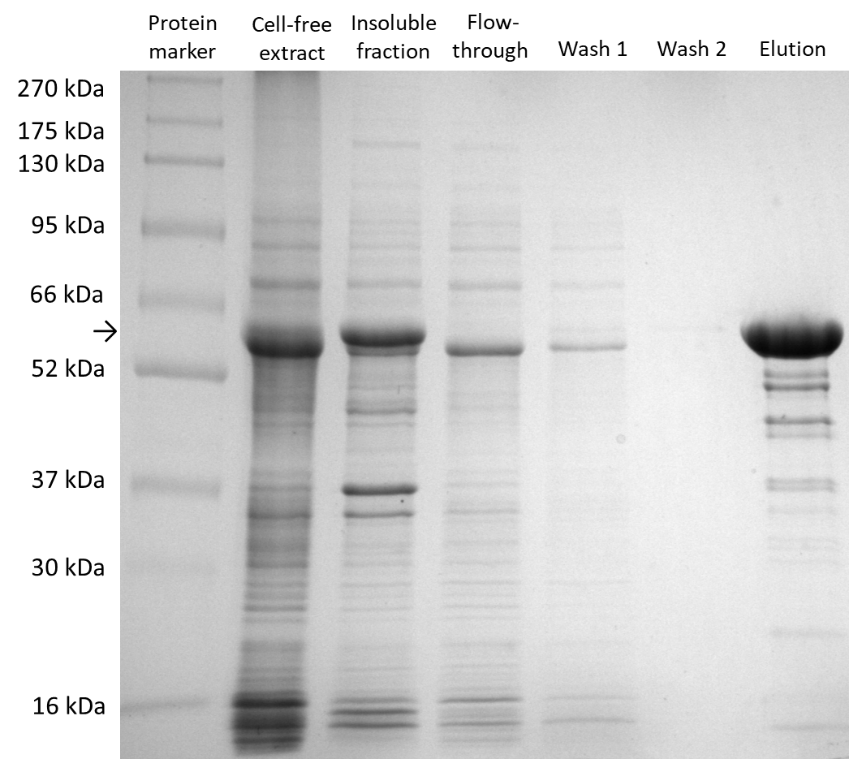


Figure S12: SDS-PAGE of MsAA NTag overexpression and purification from E. coli BL21 (DE3) with GroEL/S co-expression.

Lane 1: Protein marker (BlueEasy Prestained Protein Marker, Nippon Genetics); lane 2: cell-free extract with MsAA NTag; lane 3: insoluble fraction with MsAA NTag; lane 4: Flow-through from purification; lane 5: first wash fraction; lane 6: second wash fraction; lane 7: Elution of MsAA NTag.

MALDI-TOF analysis:


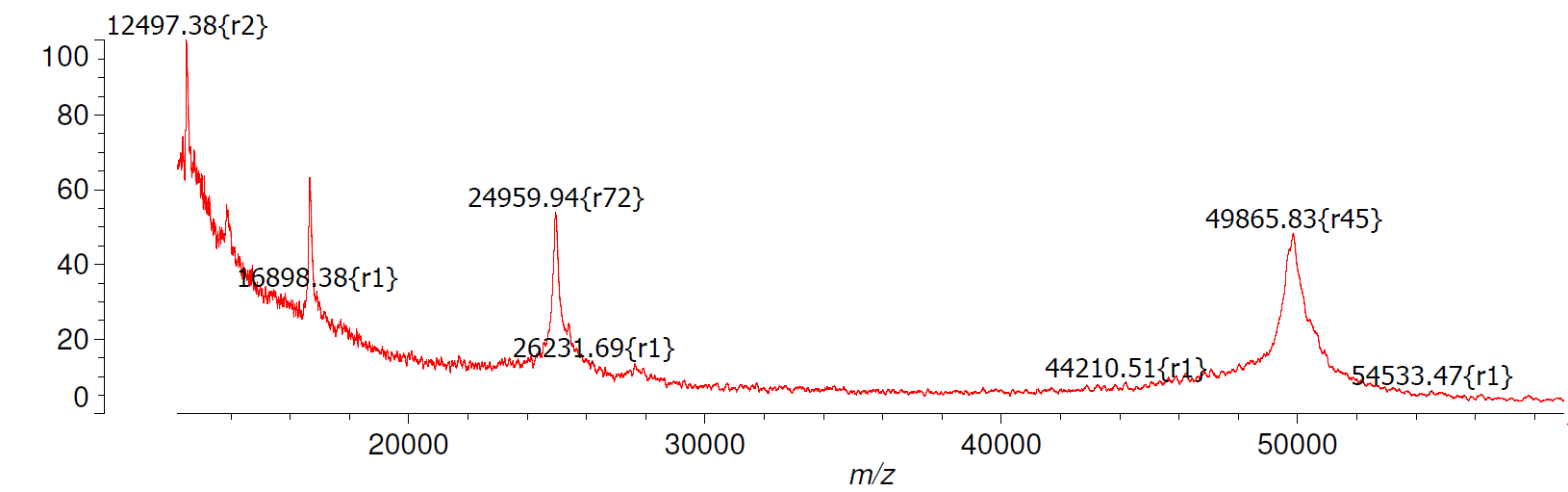


Intensity [%]

Figure S13: MALDI-TOF mass spectrum of purified MsAA NTag

The relative intensity is plotted against the m/z-values. The numbers above the peaks indicate the measured m/z-value.

Native PAGE:


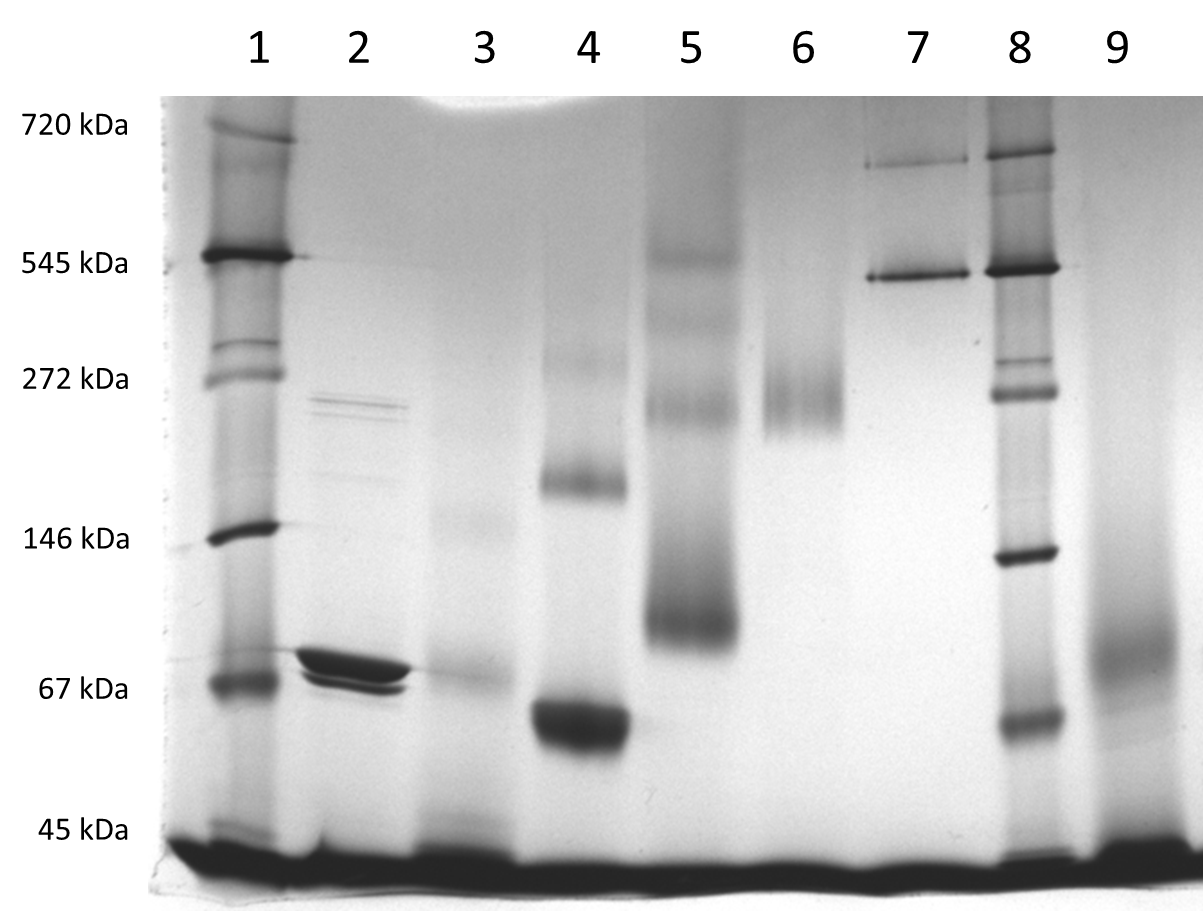


Figure S14: Native PAGE of MsAA and reference proteins.

lane 1: protein marker (SERVA Native Marker, Liquid Mix for BN/CN); lane 2: MsAA; lane 3: ovalbumin (44 kDa); lane 4: BSA (67 kDa); lane 5: conalbumin (75 kDa); lane 6: aldolase (158 kDa); lane 7: ferritin (440 kDa); lane 8: protein marker; lane 9: CsAga (Alpha-glutamine aminoacylase from Corynebacterium striatum Ax20; 96 kDa dimeric/48 kDa monomeric; heterologously expressed; unpublished data)

Isoelectric focusing:


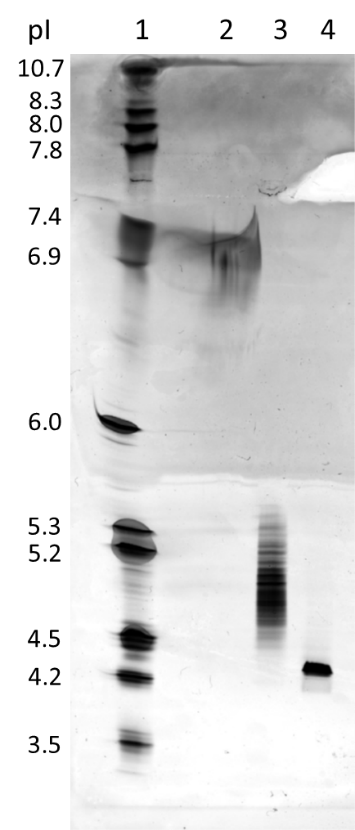


Figure S15: Isoelectric focusing for pI analysis of MsAA NTag.

Lane 1: protein marker (IEF Marker 3-10, Liquid Mix, Serva); lane 2 and 3: other protein samples; lane 4: MsAA


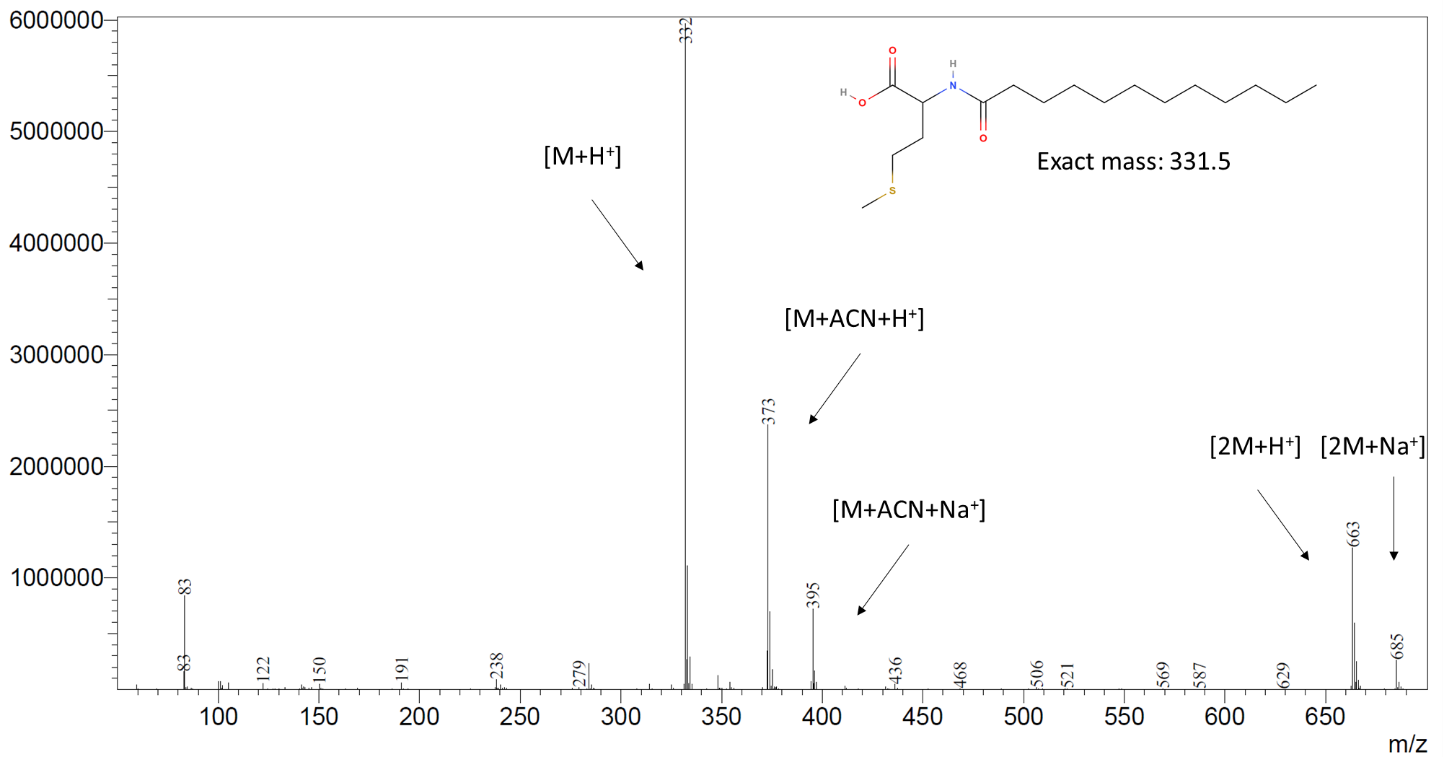


Figure S16: Mass-spectrum of N-lauroyl-L-methionine produced by MsAA
